# Synthesis of Demissidine Analogues from Tigogenin via Imine Intermediates †

**DOI:** 10.3390/ijms221910879

**Published:** 2021-10-08

**Authors:** Agnieszka Wojtkielewicz, Urszula Kiełczewska, Aneta Baj, Jacek W. Morzycki

**Affiliations:** Faculty of Chemistry, University of Białystok, K. Ciołkowskiego 1K, 15-245 Białystok, Poland; ulakielczewska@interia.eu (U.K.); aneta.baj@uwb.edu.pl (A.B.)

**Keywords:** steroidal alkaloids, solanidane alkaloids, demissidine, solanidine

## Abstract

A five-step transformation of a spiroketal side chain of tigogenin into an indolizidine system present in solanidane alkaloids such as demissidine and solanidine was elaborated. The key intermediate in the synthesis was spiroimine **3** readily obtained from tigogenin by its RuO_4_ oxidation to 5,6-dihydrokryptogenin followed by amination with aluminum amide generated in situ from DIBAlH and ammonium chloride. The mild reduction of spiroimine to a 26-hydroxy-dihydropyrrole derivative and subsequent mesylation resulted in the formation of 25-epidemissidinium salt or 23-sulfone depending on reaction conditions.

## 1. Introduction

**Figure 1:**
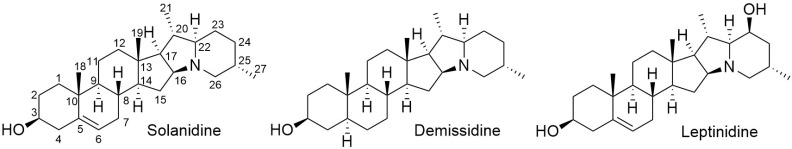
Steroidal alkaloids of solanidane type.

Demissidine and solanidine are the main representatives of the solanidane alkaloids that occur mainly as glycosides in potato species including *Solanum tuberosum*, *Solanum demissum*, and *Solanum acaule* (Figure 1) [1,2]. The various biological properties of these cholestane alkaloids have been reported in the literature [3]. Among these, α-solanine and α-chaconine, two main solanidine glycosides, are potent enough to inhibit proliferation and induce apoptosis in various types of cancer cells including cervical, liver, lymphoma, and stomach cancer cells [4]. The effectiveness of α-chaconine against hepatocellular cancer HepG2 cells is higher than the common anticancer agents doxorubicin and camptothecin [5]. Additionally, demissidine and its natural glycoside, commersonine, inhibit the growth of human colon and liver cancer cells in culture [5]. Apart from showing antitumor activity, solanidane-type alkaloids are known to act as natural insect deterrents, have antimicrobial and anti-inflammatory properties, inhibit acetylcholinesterase, and disrupt cell membranes [3,6,7,8,9]. Additionally, studies of solanidine and demissidine analogues confirm their potency for the design of new pharmacologically active agents [10,11,12].

So far, eight syntheses of solanidine and demissidine have been described, four of them in the last decade, and the latest one was reported last year [13,14,15,16,17,18,19,20]. Although recently invented methods brought a significant improvement, the described methods suffer from several drawbacks, such as multi-step procedures or unsatisfactory yields. Moreover, they cannot be easily adapted to the synthesis of demissidine or solanidine analogues. Therefore, the elaboration of an efficient route to demissidine congeners is still needed. An improved approach to the synthesis of different demissidine stereoisomers has been recently reported [21]. Here, we propose an alternative strategy toward demissidine analogues from an easily available steroid sapogenin—tigogenin.

## 2. Results and Discussion

**Scheme 1:**
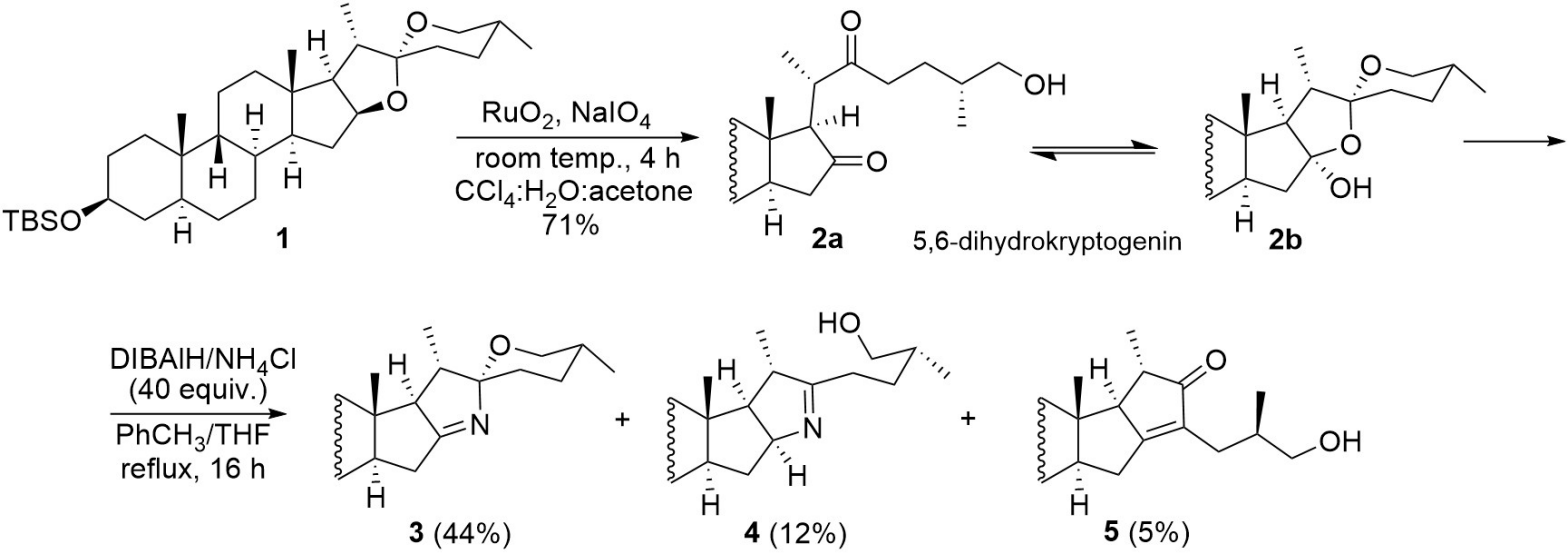
Synthesis of spiroimine **3** from tigogenin 3-TBS ether (**1**).

We found that a convenient intermediate for the transformation of the spiroketal system present in steroidal sapogenins, e.g., tigogenin, into the solanidane framework of demissidine was spiroimine **3**, shown in Scheme 1. This novel spirostane aza-analogue was obtained from tigogenin by a two-step protocol involving tigogenin oxidation to a 5,6-dihydrokryptogenin derivative and its reaction with aluminum amide as an aminating agent.

The most convenient method for the oxidative cleavage of sapogenin spiroketal to hydroxy-diketone was chosen first. After perusing the known literature protocols [22,23,24,25], we employed the RuO_4_/NaIO_4_ catalytic system. The desired 5,6-dihydrokryptogenin derivative was obtained by the oxidation of tigogenin 3-TBS ether (**1**) as a mixture of two tautomers **2a** and **2b** in 71% yield. In the next step, the obtained product was subjected to a reaction with aluminum amide generated in situ from diisobutylaluminum hydride (DIBAlH) and ammonium salt. The use of various aminoalanes as aminating agents for such compounds as epoxides, ketones, carboxylic acids, and their derivatives (chlorides, esters) has previously been widely reported in the literature [26,27,28,29,30,31]. Our previous investigations have shown that the desired aminoalane might be readily synthesized by the treatment of DIBAlH with ammonium chloride under mild reaction conditions (0 °C – room temperature, THF, up to 2 h) [32]. However, the reagent proved to be unstable and its structure was not definitely determined. The reaction of the **2a**/**2b** mixture with aminoalane prepared as described above was carried out in refluxing THF/toluene (Scheme 1). Spiroimine **3** was obtained as the main reaction product (44%) when using aluminum amide prepared in situ from 40 equivalents of DIBAlH and 42 equivalents of ammonium chloride. It is worth noting that this compound was not formed in the absence of DIBAlH. Compound **3** was accompanied by two minor products, imine **4** (12%) and enone **5** (5%). Ketone **5** was produced as a result of an aldol condensation probably due to enolization caused by aluminum amide playing a role as a Lewis acid. The formation of imine **4** in the experiment was unexpected and difficult to explain in terms of the substrate **2a**/**2b** reaction with prepared aminoalane. It seems that some unreacted diisobutylaluminum hydride was still present in the reaction mixture, resulting in the reduction of initially produced spiroimine **3** to **4** (vide infra). The reaction of compounds **2a** and **2b** with aminoalane prepared from a lower amount of DIBAlH led to the incomplete conversion. For example, employing the aminating reagent produced from 20 equivalents of DIBAlH, imine **3** was obtained in 26% yield only, while the α,β-unsaturated ketone **5** was produced in 25% yield. In this case, compound **4** was not isolated. Both imines, **3** and **4**, appeared to be convenient substrates for the synthesis of solasodine or solanidine derivatives. The mild reduction of spiroimine should provide hitherto unknown ‘reverse’ spirosolanes with the nitrogen atom in the pyrrolidine E-ring and the oxygen atom in the ‘pyranose’ F-ring. Moreover, the reductive cleavage of the spiroimine F-ring may open a direct way to solanidane alkaloids possessing an indolizidine moiety. 

**Scheme 2:**
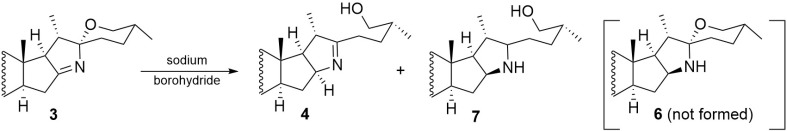
Reduction of imine **3** with borohydrides.

First, the reduction of compound **3** under mild conditions was attempted. Interestingly, the expected hemiaminal **6** (Scheme 2) was not obtained, though various reducing agents were examined. Using an equimolar amount of various borohydrides, such as NaBH_4_, NaBH_4_/I_2_, and NaBH_3_CN, under different conditions (temperature, reaction times), the main isolated product was always imine **4** accompanied by small amounts of pyrrolidine **7**. The other examined reducing agents (DIBAlH, H_2_/PtO_2_, H_2_/Pd, Hantzsch ester/TFA [33], TESH/acid) proved less effective.

**Figure 2:**
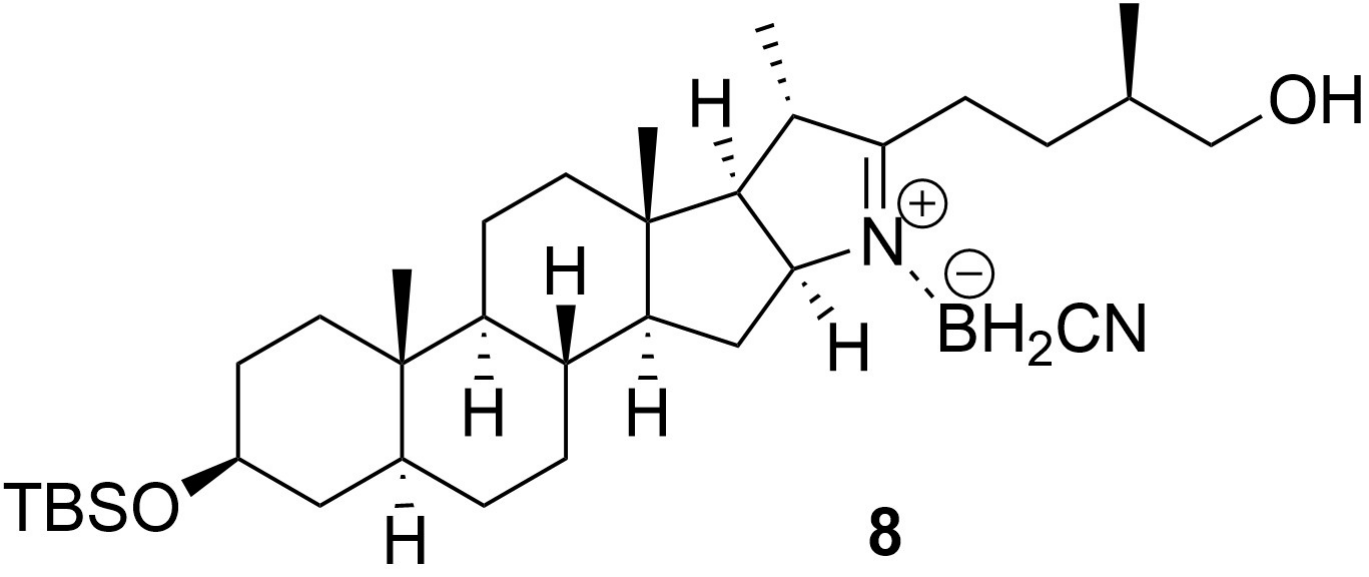
Complex **8** formed during the imine **3** reduction with NaBH_3_CN/AcOH.

The above-described results of the reduction experiments pointed out that compound **6** is less stable than its open-chain isomer **4** (confirmed by calculations). This explains unsuccessful attempts of imine **4** cyclization to **6** in the presence of acids. The observed behavior of imine **4** is clearly different from that of ‘pseudosapogenins’, which readily cyclize to spiroketals. The latter are relatively stable compounds, though their F-ring opening occurs when they are treated with strong Lewis acids. The natural aza-analogues of spirostanes (spirosolanes) containing the nitrogen atom in ring F, e.g., solasodine or tomatidine, are even more susceptible to an electrophilic attack than spiroketals. However, in the case of spirosolanes, the ‘furanose’ E-ring is readily opened [34]. This is because the cation resulting from the C–O bond cleavage is stabilized by electrons of the neighboring nitrogen atom. It seems that the isomeric compounds containing the nitrogen atom in the E-ring undergo the opening of the ‘pyranose’ F-ring for the same reason. The cleavage of the oxygen-containing ring in spirosolanes was also observed under the reducing conditions [35,36]. Despite the failure to obtain a ‘reverse’ spirosolane analogue from imine **3**, it still seemed to be a convenient intermediate for the synthesis of solanidane alkaloids. A derivative of imine **4** was previously employed by Uhle and Tian to build an indolizidine system. In the solanidine analogue synthesis reported by Uhle [37], the imine was obtained in 20% yield from kryptogenin 16-(2,4-dinitrophenyl)hydrazone and transformed into 25-episolanidine by refluxing with KOH in ethylene glycol in 65% yield. In 2016, Tian and coworkers [18] developed a new way to synthesize solanidine and demissidine using diosgenin or tigogenin as a starting material, respectively. In the method proposed by the Chinese group, 26-methyl ester 22-imine was prepared in five steps and further transformed into the desired alkaloid by the selective reduction of the imine moiety to pyrrolidine, spontaneous intramolecular aminolysis of the obtained amino-ester to lactam, and reduction. The use of spiroimine **3** as an intermediate for the construction of an indolizidine unit allowed us to shorten the solanidane synthesis from tigogenin. The approach explored in our study involved the reduction of spiroimine **3** to dihydropyrrole **4** followed by its cyclization and reduction. As our initial studies on the imine reduction showed that only complex borohydrides were effective for this transformation, we went on to optimize the reduction reaction conditions using NaBH_4_ and NaBH_3_CN as reducing agents. Our results are summarized in Table 1. Apart from compound **4**, in most cases a small amount of amine **7** was formed. Imine **4** was obtained in the best yield in reaction with a NaBH_4_/I_2_ system (entry 5). With 1 equivalent of NaBH_4_ (without any additives or with AcONa) at a low temperature and controlling the reaction time, we restrained imine over-reduction and obtained compound **4** in good yield (entry 2, 3, 4). NaBH_3_CN was less efficient (entry 6, 7). Additionally, when NaBH_3_CN was used in the presence of AcOH, an imine–cyanoborane complex **8** was formed (Figure 2).

**Scheme 3:**
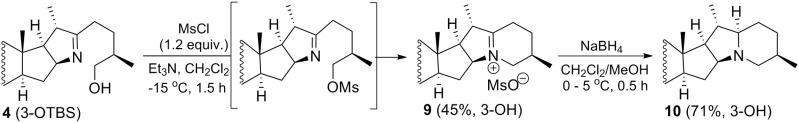
Synthesis of 25-epidemissidine (**10**).

We envisaged that the activation of the 26-hydroxyl group in compound **4** would result in spontaneous ring closing to iminium salt. Therefore, we subjected compound **4** to a reaction with mesyl chloride. As examples of the successful chemoselective mesylation of the primary hydroxyl group in the presence of amine function could be found in the literature [38,39,40], we supposed that the chemoselective mesylation of hydroxy-imine should be reached under similar conditions. The initial mesylation of hydroxy-imine **4** carried out with 1.2 equivalents of mesyl chloride in the presence of triethylamine at −15 °C resulted mainly in a less polar product (26-mesyloxy-imine), which spontaneously cyclized after work-up to the desired iminium salt **9** (Scheme 3). Under mesylation conditions, TBS protection of the 3-OH group was also removed and the indolizinium salt **9** was isolated in 45% yield. Compound **9** was readily transformed into 25-epidemissidine (**10**) by reduction with NaBH_4_. 

**Figure 3:**
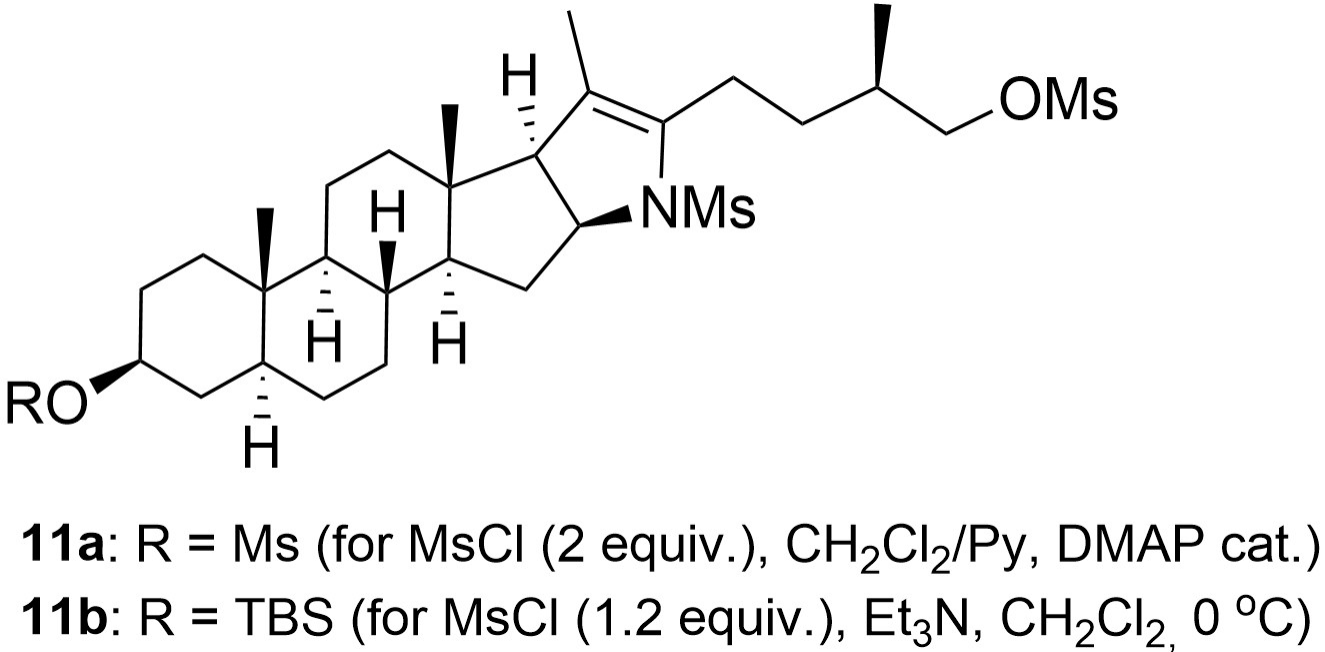
Major products of imine **4** mesylation under harsh conditions.

Conducting the mesylation under slightly harsher conditions (1.2 equiv. of MsCl, Et_3_N, 0 °C or 2 equiv. of MsCl, Py, DMAP(cat.), 0 °C–room temp.) led to a complex mixture of products. The iminium salt was formed only in 5% yield, while the main reaction product was identified as an enamine *N*,*O*-dimesyl derivative **11a** or **11b** (Figure 3).

**Scheme 4:**
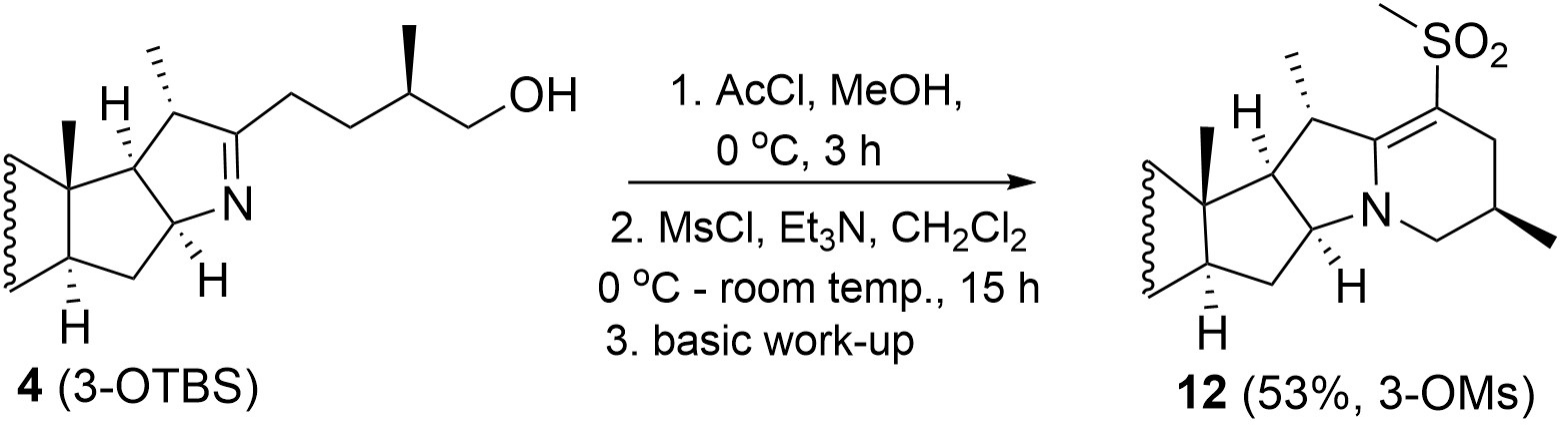
Mesylation of imine **4** preceded by protonation with HCl.

As the changes made did not result in the yield improvement of the desired indolizinium salt, we also attempted to improve the chemoselectivity of *O*-mesylation by deactivating the imine nitrogen. For this purpose, hydroxy-imine **4** was reacted with hydrogen chloride (generated in situ from AcCl and MeOH) to obtain imine hydrochloride before mesylation. The crude salt without isolation was subjected to mesylation with 2 equivalents of MsCl in the presence of Et_3_N at 0 °C–room temp. To our surprise, after basic work-up sulfone **12** (Scheme 4) was isolated, instead of the expected indolizinium salt **9**. The obtained solanidane seems to be a valuable intermediate for the synthesis of leptinidine analogues. 

**Scheme 5:**
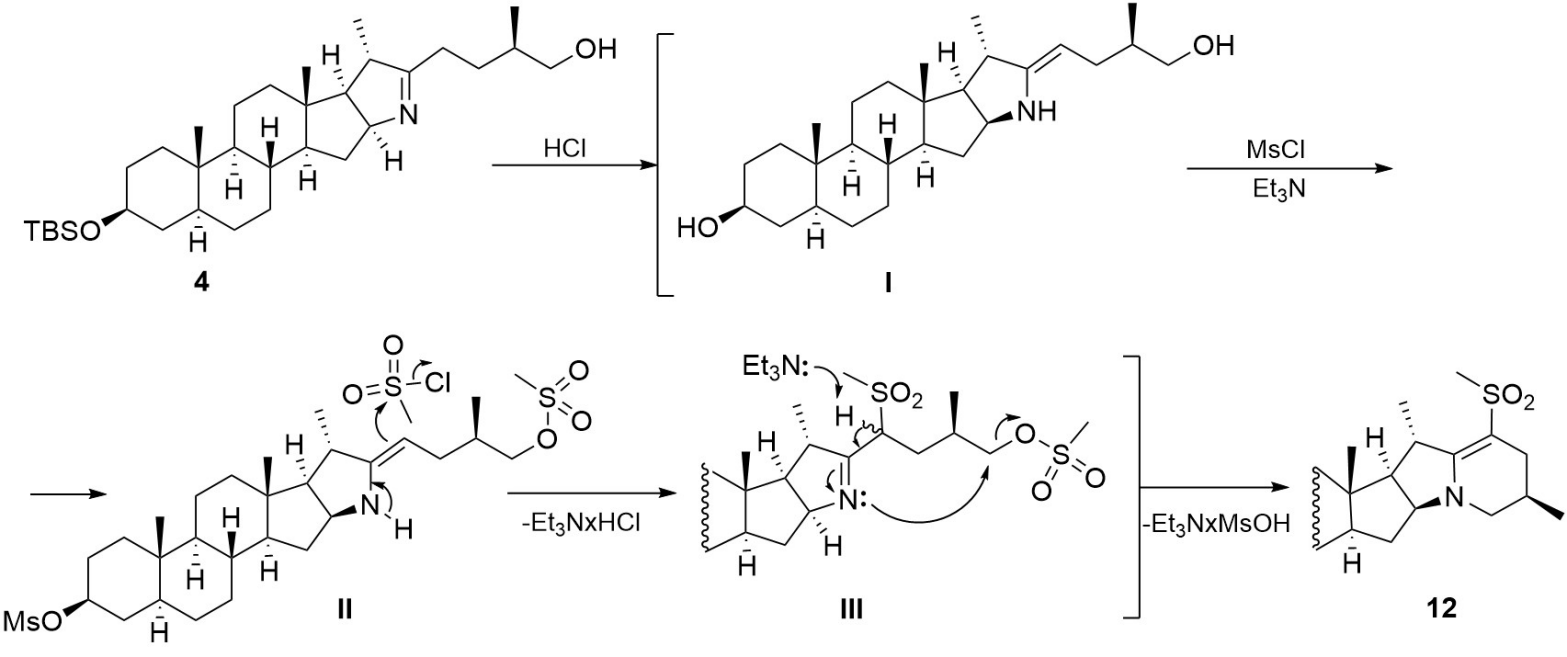
Tentative mechanism of sulfone **12** formation from imine **4**.

The hypothetical mechanism of sulfone formation is outlined in Scheme 5. An addition of HCl caused the tautomerization of imine to enamine (I) via the in situ formation of iminium salt and simultaneous deprotection of TBS ether. The enamine (I) possessing three nucleophilic sites, primary OH group, secondary OH group, and enamine carbon atom, reacted further with mesyl chloride. Apart from alcohol mesylation (II), the mesylation of an enamine electron-rich carbon occurred, leading to sulfone formation with the reconstruction of imine in ring E (III). In the final step, the cyclization to indolizine took place via an intramolecular nucleophilic substitution of 26-mesylate with the imine nitrogen. The sequence of the last-mentioned transformations (the sulfone formation followed by the ring closing) is not obvious. The reverse order of transformations (with the cyclization first) is less likely but could not be excluded. It should be mentioned that a small amount of sulfone was also formed in the mesylation of imine **4** without pre-addition of HCl.

The novel compounds prepared within the study, including the imine intermediates that frequently show antibiotic activity [41], will be subjected to biological activity evaluation in due course.

## 3. Materials and Methods

### 3.1. General 

NMR spectra were recorded with Bruker Avance II 400 spectrometer operating at 400 MHz, using CDCl_3_ solutions with TMS as the internal standard (only selected signals in the ^1^H NMR spectra are reported). Coupling constants (*J*) are given in Hz. The spectra of compounds 3–10 and 12 are included in the Supplementary Materials. The FTIR spectra were obtained using Nicolet™ 6700 spectrometer (Thermo Scientific, Waltham, MA, USA). The spectra were recorded in the range between 4000 and 500 cm^−1^ with a resolution of 4 cm^−1^ and 32 scans using Attenuated Total Reflectance (ATR) techniques. ESI and ESI-HRMS spectra were obtained on the Agilent 6530 Accurate-Mass Q-TOF ESI and LC/MS system. Melting points were determined using MP70 Melting Point System (Mettler Toledo, Greifensee, Switzerland). Thin-layer chromatography (TLC) was performed on aluminum plates coated with silica gel 60 F254 (Merck, Darmstadt, Germany), by spraying with ceric ammonium molybdate (CAM) solution, followed by heating. The reaction products were isolated by column chromatography, performed using 70–230 mesh silica gel (J. T. Baker). 

### 3.2. Chemical Synthesis

#### 3.2.1. Oxidation of 3-TBS Tigogenin (**1**) with RuO_4_/NaIO_4_

Solution of NaIO_4_ (1.8 g, 8.4 mmol) and RuO_2_ (23 mg, 0.17 mmol) in the mixture of water (20 mL), acetone (10 mL), and tetrachloride (20 mL) was vigorously stirred until the yellow color of RuO_4_ appeared. Then, a solution of 3-TBS tigogenin (**1**, 0.3 g, 0.57 mmol) in 8 mL of CCl_4_ was added in three portions and the reaction mixture was stirred for 10 h at room temperature. After that time, the TLC control showed that no starting material remained. A few drops of isopropanol were added to quench RuO_4_ and the resulting slurry was stirred for an additional 10 min at room temperature (yellow RuO_4_ turned into black RuO_2_). The reaction mixture was poured into water and product was extracted with CHCl_3_. The extract was dried over anhydrous sodium sulfate, and the solvent was evaporated. Silica gel column chromatography afforded the product as an equilibrium mixture of two tautomers **2a** and **2b**, identical to that described in reference [42] in 73% total yield. 

Compound **2a**/**2b**, eluted with 7.5% to 25% AcOEt/hexane: for main tautomer: ^1^H NMR (400 MHz, CDCl_3_) δ 3.58 (m, 2H), 3.47 (m, 1H), 2.57 (m, 1H), 2.62 (m, 1H), 1.02 (d, *J* = 7.0, 3H), 0.95 (d, *J* = 6.6, 3H), 0.89 (s, 9H), 0.81 (s, 3H), 0.74 (s, 3H), 0.06 (s, 6H); ESI-MS 547 [M+H]^+^. HRMS calculated for C_33_H_59_O_4_Si (M+H)^+^, 547.4177; found 547.4230. 

#### 3.2.2. Synthesis of (25*R*)-3β-*t*-butyldimethylsililoxy-16-aza-spirost-16(*N*)-ene (**3**)

*Preparation of the aminoalane reagent from DIBALH and NH_4_Cl*

A solution of DIBAlH in toluene (1 M, 22 mL, 22 mmol, 40 equiv. relative to compounds **2a** and **2b**) was added to a cooled (0–5 °C) suspension of NH_4_Cl (1.23 g, 23 mmol, 42 equiv.) in anhydrous THF (15 mL) under argon. The reaction was stirred for 15 min in an ice bath and then 1.5 h at room temperature. After this time, the obtained reagent solution was used directly for the reaction with compound **2a**/**2b**.

*Synthesis of imine **3***

The solution of aminoalane reagent (prepared from 40 equiv. of DIBAlH) was added dropwise to a solution of compound **2a** and **2b** (0.3 g, 0.549 mmol) in anhydrous THF (ca 6 mL) at room temperature. Then, stirring was continued for 16 h at reflux. After this time, the reaction mixture was cooled, quenched with aqueous solution of KHSO_4_, and the product was extracted with ether. The extract was washed with water, dried over anhydrous sodium sulfate, and the solvent was evaporated. Silica gel column chromatography afforded three products: spiroimine **3** (44%) eluted with 10% AcOEt/hexane, α,β-unsaturated ketone **5** (5%) eluted with 15% AcOEt/hexane, and dihydropyrrole **4** (12%) eluted with 70% AcOEt/hexane.

Compound **3**: ^1^H NMR (400 MHz, CDCl_3_) δ 3.56 (m, 2H), 3.45 (dd, *J* = 11.0, 10.9, 1H), 2.56 (m, 1H), 2.45 (m, 1H), 1.01 (d, *J* = 6.9, 3H), 0.89 (s, 9H), 0.84 (d, *J* = 6.6, 3H), 0.83 (s, 3H), 0.61 (s, 3H), 0.06 (s, 6H); ^13^C NMR (100 MHz, CDCl_3_) δ 193.2 (C), 107.6 (C), 72.1 (CH), 70.6 (CH), 69.1 (CH_2_), 56.8 (CH), 54.5 (CH), 45.0 (CH), 42.7 (CH), 39.8 (C), 38.6 (CH_2_), 38.3 (CH_2_), 37.0 (CH_2_), 35.7 (C), 35.1 (CH), 33.7 (CH_2_), 32.3 (CH_2_), 31.9 (CH_2_), 30.8 (CH), 29.3 (CH_2_), 28.7 (CH_2_), 28.5 (CH_2_), 25.9 (3xCH_3_), 20.9 (CH_2_), 18.3 (C), 17.2 (CH_3_), 13.5 (CH_3_), 12.43 (CH_3_), 12.39 (CH_3_), −4.6 (2xCH_3_); ESI-MS 528 [M+H]^+^. HRMS calculated for C_33_H_59_NO_2_Si (M+H)^+^, 528.4231; found 528.4297; IR ATR, ν_max_ (cm^−1^): 1728, 1667, 1457, 1373, 1248, 1173, 1063.

Compound **4**: ^1^H NMR (400 MHz, CDCl_3_) δ 4.43 (m, 1H), 3.55 (m, 1H), 3.43 (dd, *J* = 11.1, 4.0, 1H), 3.30 (dd, *J* = 11.1, 6.0, 1H), 2.61 (q, *J* = 7.3, 1H), 2.29 (m, 2H), 2.22 (m, 1H), 1.08 (d, *J* = 7.3, 3H), 0.92 (d, *J* = 6.3, 3H), 0.89 (s, 9H), 0.79 (s, 3H), 0.51 (s, 3H), 0.05 (s, 6H); ^13^C NMR (100 MHz, CDCl_3_) δ 182.0 (C), 74.8 (CH), 72.1 (CH), 65.9 (CH_2_), 61.6 (CH), 54.9 (CH), 54.6 (CH), 45.0 (CH), 44.5 (CH), 41.4 (C), 39.2 (CH_2_), 38.6 (CH_2_), 37.2 (CH_2_), 35.9 (CH), 35.6 (C), 35.1 (CH), 32.4 (CH_2_), 32.0 (CH_2_), 31.9 (CH_2_), 28.7 (CH_2_), 27.6 (CH_2_), 27.5 (CH_2_), 25.9 (3xCH_3_), 20.8 (CH_2_), 18.8 (CH_3_), 18.3 (C), 17.0 (CH_3_), 14.0 (CH_3_), 12.4 (CH_3_), −4.6 (2xCH_3_); ESI-MS 530 [M+H]^+^. HRMS calculated for C_33_H_60_NO_2_Si (M+H)^+^, 530.4388; found 530.4398; IR ATR, ν_max_ (cm^−1^): 3235, 1631, 1454, 1372, 1250, 1095, 1062.

Compound **5**: ^1^H NMR (400 MHz, CDCl_3_) δ 3.57 (m, 1H), 3.47 (m, 1H), 3.37 (m, 1H), 3.12 (m, 1H), 2.58 (m, 1H), 2.39 (dd, *J* = 13.5, 5.6, 1H), 2.24 (bs, 1H), 2.17 (q, *J* = 7.2, 1H), 1.19 (d, *J* = 7.2, 3H), 0.89 (s, 9H), 0.83 (s, 3H), 0.80 (d, *J* = 6.8, 3H), 0.54 (s, 3H), 0.06 (s, 6H); ^13^C NMR (100 MHz, CDCl_3_) δ 214.9 (C), 183.9 (C), 134.1 (C), 72.0 (CH), 66.0 (CH_2_), 65.8 (CH), 55.9 (CH), 54.7 (CH), 45.0 (CH), 42.8 (CH), 41.2 (C), 38.6 (CH_2_), 37.9 (CH_2_), 37.1 (CH_2_), 35.7 (C), 35.2 (CH), 35.0 (CH), 32.2 (CH_2_), 31.9 (CH_2_), 28.5 (CH_2_), 28.0 (CH_2_), 26.2 (CH_2_), 25.9 (3xCH_3_), 20.9 (CH_2_), 18.3 (C), 16.4 (CH_3_), 14.5 (CH_3_), 12.4 (CH_3_), 12.3 (CH_3_), -4.6 (2xCH_3_); ESI-MS 529 [M+H]^+^. HRMS calculated for C_33_H_57_O_3_Si (M+H)^+^, 529.4071; found 529.4062; IR ATR, ν_max_ (cm^−1^): 3431, 1697, 1654, 1456, 1373, 1248, 1080, 834, 772.

#### 3.2.3. General Procedure for Imine **3** Reduction with Complex Sodium Hydride

To the stirred solution of imine **3** (1 equiv.) in the proper solvent, reducing agents (NaBH_4_, NaBH_3_CN) and additives (NaOAc, I_2_, AcOH) were added. The detailed reaction conditions are indicated in Table 1. The reaction mixture was monitored by TLC. The reaction mixture was poured into water and extracted with CHCl_3_. The extract was washed with water, dried over anhydrous sodium sulfate, and the solvent was evaporated. The crude products (**4**, **7**, **8**) were isolated by silica gel column chromatography. 

Compound **7**, eluted with 8% MeOH/CHCl_3_: ^1^H NMR (400 MHz, CDCl_3_) δ 3.75 (m, 1H), 3.51 (m, 2H), 3.37 (m, 1H), 2.88 (m, 1H), 2.01 (m, 1H), 1.01 (d, *J* = 6.4, 3H), 0.88 (s, 9H), 0.864 (s, 3H), 0.859 (d, *J* = 6.3, 3H), 0.80 (s, 3H), 0.05 (s, 6H); ^13^C NMR (100 MHz, CDCl_3_) δ 72.1 (CH), 70.4 (CH), 67.3 (CH_2_), 63.2 (CH), 62.2 (CH), 57.6 (CH), 54.3 (CH), 45.0 (CH), 41.2 (C), 40.0 (CH_2_), 38.6 (CH_2_), 38.4 (CH), 37.1 (CH_2_), 35.6 (C), 34.8 (CH), 34.7 (CH), 32.3 (CH_2_), 31.9 (CH_2_), 30.8 (CH_2_), 29.6 (CH_2_), 28.6 (CH_2_), 27.9 (CH_2_), 25.9 (3xCH_3_), 20.9 (CH_2_), 18.2 (CH_3_), 18.1 (C), 17.0 (CH_3_), 16.0 (CH_3_), 12.3 (CH_3_), −4.6 (2xCH_3_); ESI-MS 532 [M+H]^+^. HRMS calculated for C_33_H_62_NO_2_Si (M+H)^+^, 532.4544; found 532.4559; IR ATR, ν_max_ (cm^−1^): 3288, 1454, 1368, 1247, 1092.

Compound **8** (obtained by reduction with NaBH_3_CN, Table 1, entry 6), eluted with 45% AcOEt/hexane: ^1^H NMR (400 MHz, CDCl_3_) δ 4.66 (m, 1H), 3,62–3.50 (m, 3H), 3.07 (q, *J* = 7.4, 1H), 2.91 (dd, *J* = 12.4, 4.3, 1H), 2.48–2.35 (m, 2H), 1.83 (d, *J* = 8.4, 1H), 1.22 (d, *J* = 7.4, 3H), 1.00 (d, *J* = 6.6, 3H), 0.89 (s, 9H), 0.79 (s, 3H), 0.57 (s, 3H), 0,05 (s, 6H); ^13^C NMR (100 MHz, CDCl_3_) δ 191. 4 (C), 76.9 (CH), 72.0 (CH), 66.7 (CH_2_), 57.6 (CH), 54.4 (CH), 54.0 (CH), 44.9 (CH), 43.6 (CH), 42.3 (C), 38.52 (CH_2_), 38.49 (CH_2_), 37.2 (CH_2_), 35.8 (CH), 35.6 (C), 34.9 (CH), 32.2 (CH_2_), 31.9 (CH_2_), 31.2 (CH_2_), 28.7 (CH_2_), 28.4 (CH_2_), 27.6 (CH_2_), 25.9 (3xCH_3_), 20.5 (CH_2_), 18.5 (CH_3_), 18.2 (C), 16.3 (CH_3_), 14.6 (CH_3_), 12.3 (CH_3_), −4.57 (CH_3_), −4.59 (CH_3_); ^11^B NMR (128 MHz, CDCl_3_) δ −24.52; ESI-MS 1159 [2M+Na]^+^. IR ATR, ν_max_ (cm^−1^): 3468, 2401, 1636, 1458, 1249, 1093, 1056.

#### 3.2.4. Synthesis of Compound **9**

To a solution of **4** (19 mg, 0.036 mmol) in dichloromethane (2 mL) at −15 °C, Et_3_N (0.01 mL, 7.3 mg, 0.072 mmol) and 0.22 mL of solution of MsCl (0.03 mL) in dichloromethane (2 mL) were added, successively. The reaction mixture was continuously stirred at −15 °C for 1.5 h and quenched by adding aqueous NaHCO_3_, and the layers were separated and the aqueous layer was extracted with chloroform. The organic layers were combined, dried over Na_2_SO_4_, and evaporated under reduced pressure. The crude product was purified by column chromatography (20% MeOH/CHCl_3_) to obtain compound **9** (45%).

Compound **9**: ^1^H NMR (400 MHz, CDCl_3_) δ 5.45 (m, 1H), 4.25 (m, 1H), 3.61 (m, 1H), 3.13-3.00 (m, 3H), 2.75 (s, 3H), 2.72 (m, 1H), 2.62 (m, 1H), 2.32 (m, 1H), 2.18 (d, *J* = 6.1, 1H), 1.57 (d, *J* = 7.1, 3H), 1.08 (d, *J* = 5.9, 3H), 0.81 (s, 3H), 0.62 (s, 3H); δ ^13^C NMR (100 MHz, CDCl_3_) δ 190.6 (C), 75.7 (CH), 71.0 (CH), 57.0 (CH), 54.3 (CH), 54.0 (CH), 52.7 (CH_2_), 44.6 (CH), 44.5 (CH), 42.0 (C), 39.4 (CH_3_), 38.2 (CH_2_), 38.0 (CH_2_), 36.9 (CH_2_), 35.5 (C), 35.0 (CH), 32.0 (CH_2_), 31.3 (CH_2_), 29.2 (CH_2_), 28.3 (CH_2_), 25.84 (CH), 25.82 (CH_2_), 24.6 (CH_2_), 20.5 (CH_2_), 18.2 (CH_3_), 18.1 (CH_3_), 14.6 (CH_3_), 12.3 (CH_3_); ESI-MS 398 [M^+^]; IR ATR, ν_max_ (cm^−1^): 3377, 1664, 1628, 1456, 1195, 1043.

#### 3.2.5. Synthesis of 25-epidemissidine (**10**)

To the stirred ice-cooled solution of compound **9** (20 mg, 0.04 mmol) in MeOH (2 mL)/DCM (2 mL), NaBH_4_ (4.6 mg, 0.12 mmol) was added. The stirring of the reaction mixture was continued at −10 – 0 °C for 0.5 h. The reaction mixture was poured into water and extracted with CHCl_3_. The extract was washed with water, dried over anhydrous sodium sulfate, and the solvent was evaporated. The crude product was purified by column chromatography (20% AcOEt/hexane) to obtain compound **10** (71%), identical to that described in ref. [21].

Compound **10**: ^1^H NMR (400 MHz, CDCl_3_): δ 3.60 (m, 1H), 2.59 (m, 2H), 1.03 (d, *J* = 7.0, 3H), 0.92 (d, *J* = 6.7, 3H), 0.85 (s, 3H), 0.82 (s, 3H). 

#### 3.2.6. Synthesis of Compound **12**

Acetyl chloride (0.027 mL, 29 mg, 0.38 mmol) was added to the stirred, ice-cold solution of compound **4** (20 mg, 0.038 mmol) in dry MeOH (3 mL). The reaction mixture was stirred for 3 h and allowed to warm up to room temperature. Then, the solvent was evaporated under reduced pressure and the residue was dissolved in dichloromethane (2 mL) and THF (2 mL). To the obtained suspension, triethylamine (0.02 mL) and 0.58 mL of a solution of MsCl (0.02 mL) in CH_2_Cl_2_ (2 mL) were added. The reaction mixture was stirred overnight, allowing it to warm up to room temperature. After this time, the mixture was poured into aqueous NaHCO_3_ and extracted with CHCl_3_. The extract was washed with water, dried over anhydrous sodium sulfate, and the solvent was evaporated. The crude product was purified by column chromatography (35% AcOEt/hexane) to afford compound **12** (53%).

Compound **12**: ^1^H NMR (400 MHz, CDCl_3_) δ 4.62 (m, 1H), 4.04 (m, 1H), 3.61 (q, *J* = 7.0, 1H), 3.02 (m, 1H), 3.00 (s, 3H), 2.83 (s, 3H), 2.74 (m, 1H), 2.50 (d, *J* = 12.5, 1H), 1.24 (d, *J* = 7.0, 3H), 1.04 (d, *J* = 6.1, 3H), 0.83 (s, 3H), 0.61 (s, 3H); ^13^C NMR (100 MHz, CDCl_3_) δ 160.9 (C), 88.9 (C), 81.6 (CH), 65.4 (CH), 60.5 (CH), 55.0 (CH), 54.1 (CH), 49.0 (CH_2_), 44.8 (CH), 43.0 (CH_3_), 41.8 (C), 38.9 (CH_3_), 38.2 (CH_2_), 36.7 (CH_2_), 36.6 (CH), 35.3 (C), 35.1 (CH_2_), 34.9 (CH), 32.0 (CH_2_), 30.8 (CH_2_), 30.7 (CH_2_), 28.6 (CH_2_), 28.3 (CH_2_), 26.8 (CH), 23.0 (CH_3_), 20.5 (CH_2_), 18.7 (CH_3_), 13.5 (CH_3_), 12.1 (CH_3_); ESI-MS 554 [M+H]^+^, 1129 [2M+Na] ^+^. HRMS calculated for C_29_H_48_NO_5_S_2_ (M+H)^+^ 554.2968; found 554.2965; IR ATR, ν_max_ (cm^−1^): 1658, 1453, 1333, 1212, 1163, 1036, 925.

## 4. Conclusions

In summary, we developed a novel, concise synthesis of solanidanes from the spirostane sapogenin tigogenin. The indolizidine moiety present in solanidane-type alkaloids was constructed from spirostane in five steps involving tigogenin oxidation, amination, reduction, mesylation, and reduction again. The key intermediate for the proposed approach was spiroimine obtained in the reaction of a 5,6-dihydrokrytogenin derivative with aminoalane generated in situ from DIBAlH and NH_4_Cl. Depending on mesylation conditions, two different solanidanes were obtained: the indolizinium salt **9a**, which was readily converted into 25-epidemissidine (**10**), and the 23-sulfone derivative **12**, a convenient intermediate for further derivatization.

## Figures and Tables

**Table 1:** The optimization of imine **3** reduction conditions.

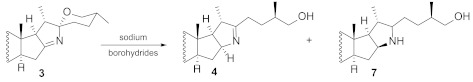					
**Entry**	**Reagent** **(equiv.)**	**Conditions**	**Product 4 Yield (%)**	**Product 7 Yield (%)**	**Substrate** **Recovery (%)**
1	NaBH_4_ (2)	MeOH/CH_2_Cl_2_, −18 °C, 1 h	64	30	<1
2	NaBH_4_ (1)	MeOH/CH_2_Cl_2_, −18 °C, 1 h	58	<5	21
3	NaBH_4_ (1)	MeOH/CH_2_Cl_2_, 0 °C, 1 h	67	13	<5
4	NaBH_4_ (1)	NaOAc (1 equiv.), MeOH/CH_2_Cl_2_, 0 °C–room temp., 2 h	65	<5	30
5	NaBH_4_ (4)	I_2_ (4 equiv.), MeOH/ CH_2_Cl_2_, 0 °C–reflux, 16 h	78	9	<1
6	NaBH_3_CN (2)	AcOH (2 equiv.), THF, room temp., 1 h	complex 8 (48)	nd *	<5
7	NaBH_3_CN (2)	THF/MeOH, room temp., 2 h	28	nd *	30

* nd—not detected.

## Supplementary Materials

The following are available online at https://www.mdpi.com/article/10.3390/ijms221910879/s1, ^1^H NMR, ^13^C NMR spectra of compounds **3**–**10** and **12**.
