# Supplementary material for: Synthesis of Demissidine Analogues from Tigogenin via Imine Intermediates
Source: Int J Mol Sci. 2021 Oct 8;22(19):10879. doi: 10.3390/ijms221910879 (PMC8509427; doi:10.3390/ijms221910879)
Supplement: Supplementary file 1 [file ijms-22-10879-s001.zip › ijms-1406804-supplementary.pdf]

Supplementary Materials for

Synthesis of demissidine analogues from tigogenin via imine intermediates†

Agnieszka Wojtkielewicz *, Urszula Kiełczewska, Aneta Baj and Jacek W. Morzycki *

Faculty of Chemistry, University of Białystok, K. Ciołkowskiego 1K, 15-245 Białystok, Poland; ulakielczewska@interia.eu (U.K.); aneta.baj@uwb.edu.pl (A.B.)

* Correspondence: a.wojtkielewicz@uwb.edu.pl, Tel.: +48-857388043 (A.W.); morzycki@uwb.edu.pl, Tel.: +48-857388260 (J.W.M.)

† Dedicated to Prof. Dr. Ludger Wessjohann on the occasion of his 60th birthday

Table of contents

Spectra of compound **3** (^{1}H NMR, ^{13}C NMR, DEPT)……………………………………………………….2

Spectra of compound **4** (^{1}H NMR, ^{13}C NMR, DEPT)……………………………………………………......5

Spectra of compound **5** (^{1}H NMR, ^{13}C NMR, DEPT)…………………………………………………..........8

Spectra of compound **7** (^{1}H NMR, ^{13}C NMR, DEPT)………………………………………………………11

Spectra of compound **8** (^{1}H NMR, ^{13}C NMR, DEPT)………………………………………………………14

Spectra of compound **9** (^{1}H NMR, ^{13}C NMR, DEPT)………………………………………………………17

Spectra of compound **10** (^{1}H NMR, ^{13}C NMR, DEPT)……………………………………………………..20

Spectra of compound **12** (^{1}H NMR, ^{13}C NMR, DEPT)……………………………………………………..23

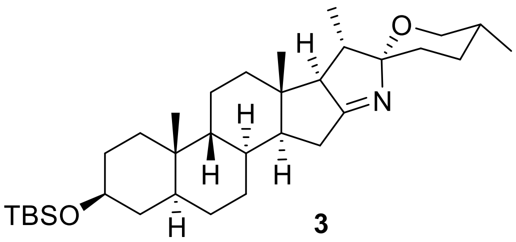
TBSO
H
H
H
H
H
O
N
3

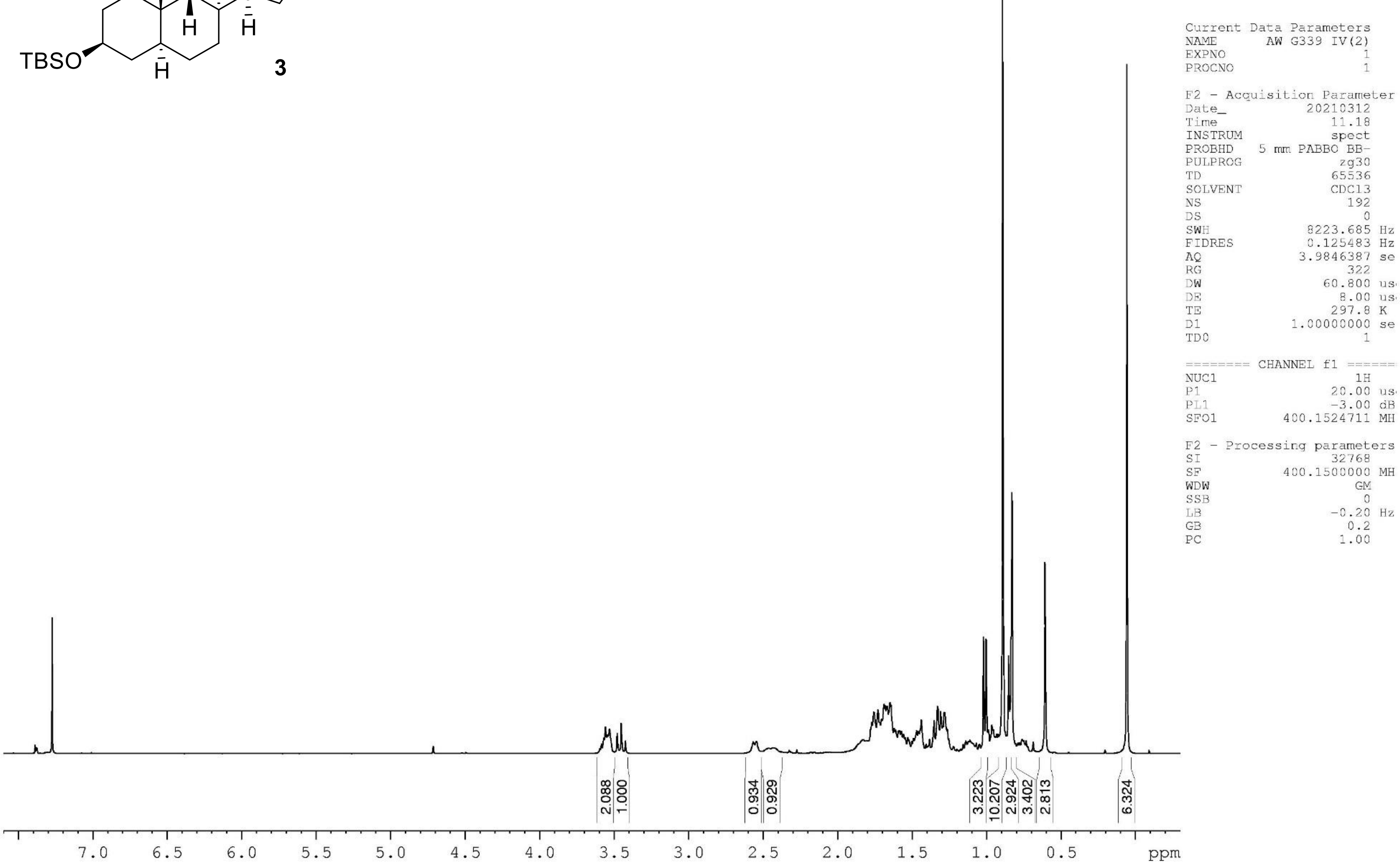
Current Data Parameters
NAME AW G339 IV(2)
EXPNO 1
PROCNO 1
F2 - Acquisition Parameter
Date_ 20210312
Time 11.18
INSTRUM spect
PROBHD 5 mm PABBO BB-
PULPROG zg30
TD 65536
SOLVENT CDCl3
NS 192
DS 0
SWH 8223.685 Hz
FIDRES 0.125483 Hz
AQ 3.9846387 se
RG 322
DW 60.800 us
DE 8.00 us
TE 297.8 K
D1 1.00000000 se
TD0 1
======== CHANNEL f1 ======
NUC1 1H
P1 20.00 us
PL1 -3.00 dB
SFO1 400.1524711 MH
F2 - Processing parameters
SI 32768
SF 400.1500000 MH
WDW GM
SSB 0
LB -0.20 Hz
GB 0.2
PC 1.00
2.088
1.000
0.934
0.929
3.223
10.207
2.924
3.402
2.813
6.324
7.0 6.5 6.0 5.5 5.0 4.5 4.0 3.5 3.0 2.5 2.0 1.5 1.0 0.5 ppm

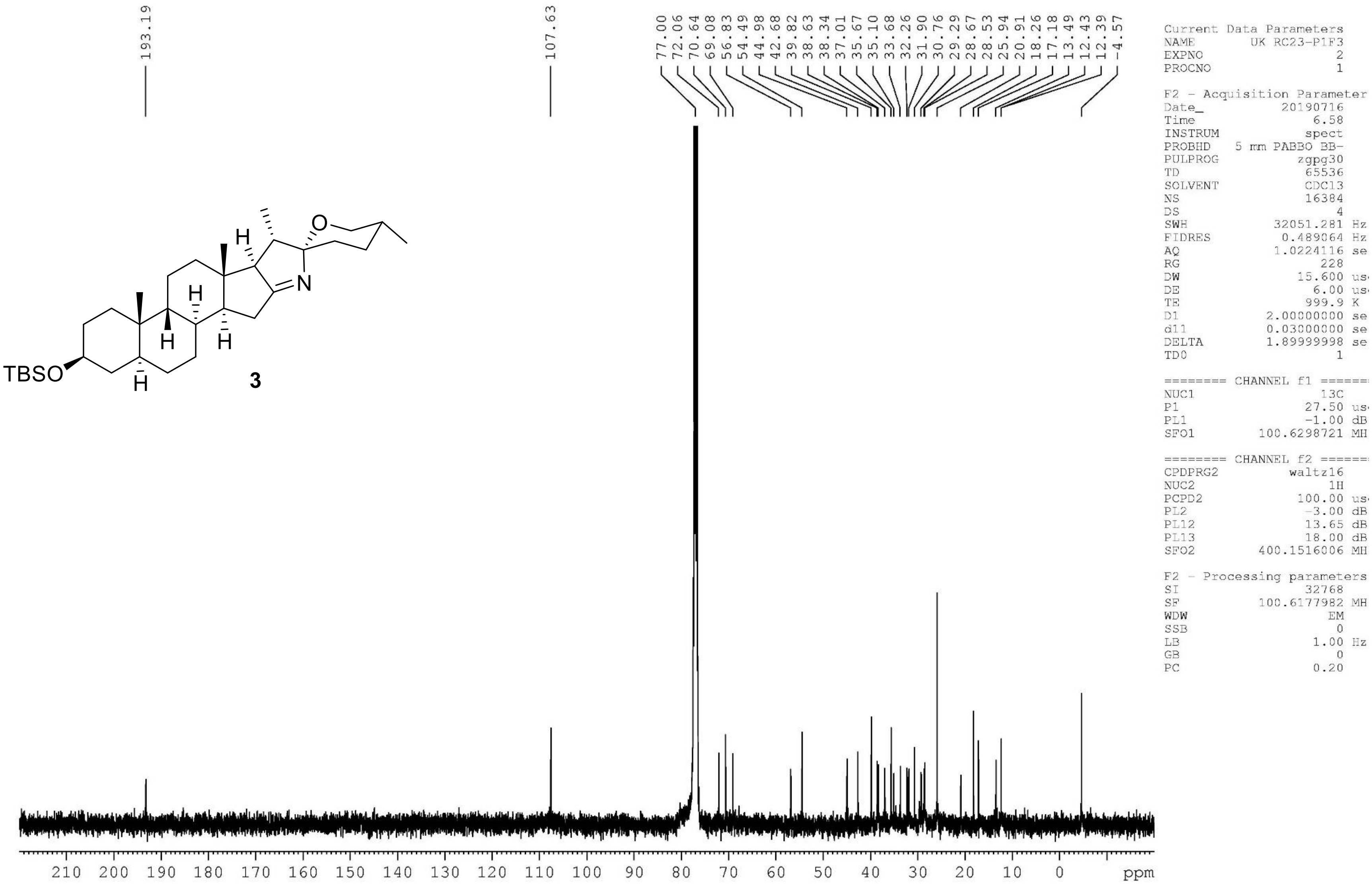
TBSO
H
H
H
H
H
O
N
3
193.19
107.63
77.00
72.06
70.64
69.08
56.83
54.49
44.98
42.68
39.82
38.63
38.34
37.01
35.67
35.10
33.68
32.26
31.90
30.76
29.29
28.67
28.53
25.94
20.91
18.26
17.18
13.49
12.43
12.39
-4.57
210 200 190 180 170 160 150 140 130 120 110 100 90 80 70 60 50 40 30 20 10 0 ppm
Current Data Parameters
NAME UK RC23-P1F3
EXPNO 2
PROCNO 1
F2 - Acquisition Parameter
Date_ 20190716
Time 6.58
INSTRUM spect
PROBHD 5 mm PABBO BB-
PULPROG zgpg30
TD 65536
SOLVENT CDCl3
NS 16384
DS 4
SWH 32051.281 Hz
FIDRES 0.489064 Hz
AQ 1.0224116 se
RG 228
DW 15.600 us
DE 6.00 us
TE 999.9 K
D1 2.00000000 se
d11 0.03000000 se
DELTA 1.89999998 se
TD0 1
======== CHANNEL f1 ======
NUC1 13C
P1 27.50 us
PL1 -1.00 dB
SFO1 100.6298721 MH
======== CHANNEL f2 ======
CPDPRG2 waltz16
NUC2 1H
PCPD2 100.00 us
PL2 -3.00 dB
PL12 13.65 dB
PL13 18.00 dB
SFO2 400.1516006 MH
F2 - Processing parameters
SI 32768
SF 100.6177982 MH
WDW EM
SSB 0
LB 1.00 Hz
GB 0
PC 0.20

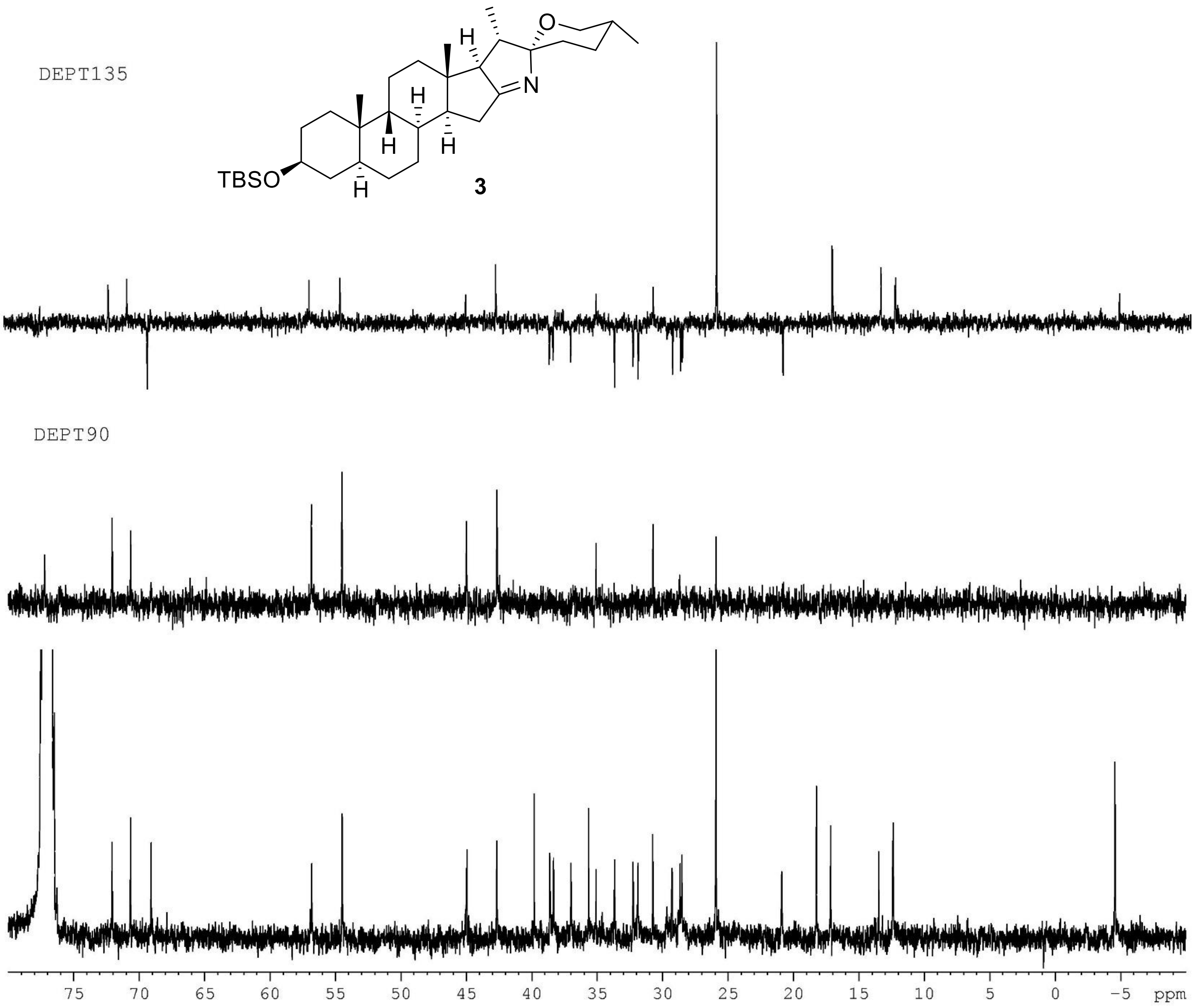


```
Current Data Parameters
NAME         UK RC23-P1F3
EXPNO                   4
PROCNO                  1

F2 - Acquisition Parameter
Date_            20190716
Time                 9.49
INSTRUM             spect
PROBHD   5 mm PABBO BB-
PULPROG          dept135
TD                  65536
SOLVENT             CDCl3
NS                   3200
DS                      4
SWH          36231.883 Hz
FIDRES        0.552855 Hz
AQ           0.9044468 se
RG                   2050
DW              13.800 us
DE                6.00 us
TE               999.9 K
CNST2        145.0000000
D1          2.00000000 se
d2          0.00344828 se
d12         0.00002000 se
DELTA       0.00003501 se
TD0                     1

======== CHANNEL f1 ======
NUC1                  13C
P1               27.50 us
p2               55.00 us
PL1              -1.00 dB
SFO1       100.6213196 MH

======== CHANNEL f2 ======
CPDPRG2          waltz16
NUC2                   1H
P3               25.00 us
p4               50.00 us
PCPD2           100.00 us
PL2              -3.00 dB
PL12             13.65 dB
SFO2       400.1516006 MH

F2 - Processing parameters
SI                  32768
SF         100.6177980 MH
WDW                    EM
SSB                     0
LB                1.00 Hz
GB                      0
PC                   0.20
```

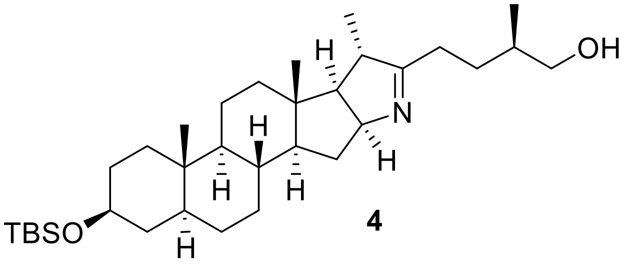

TBSO
H
N
OH

4

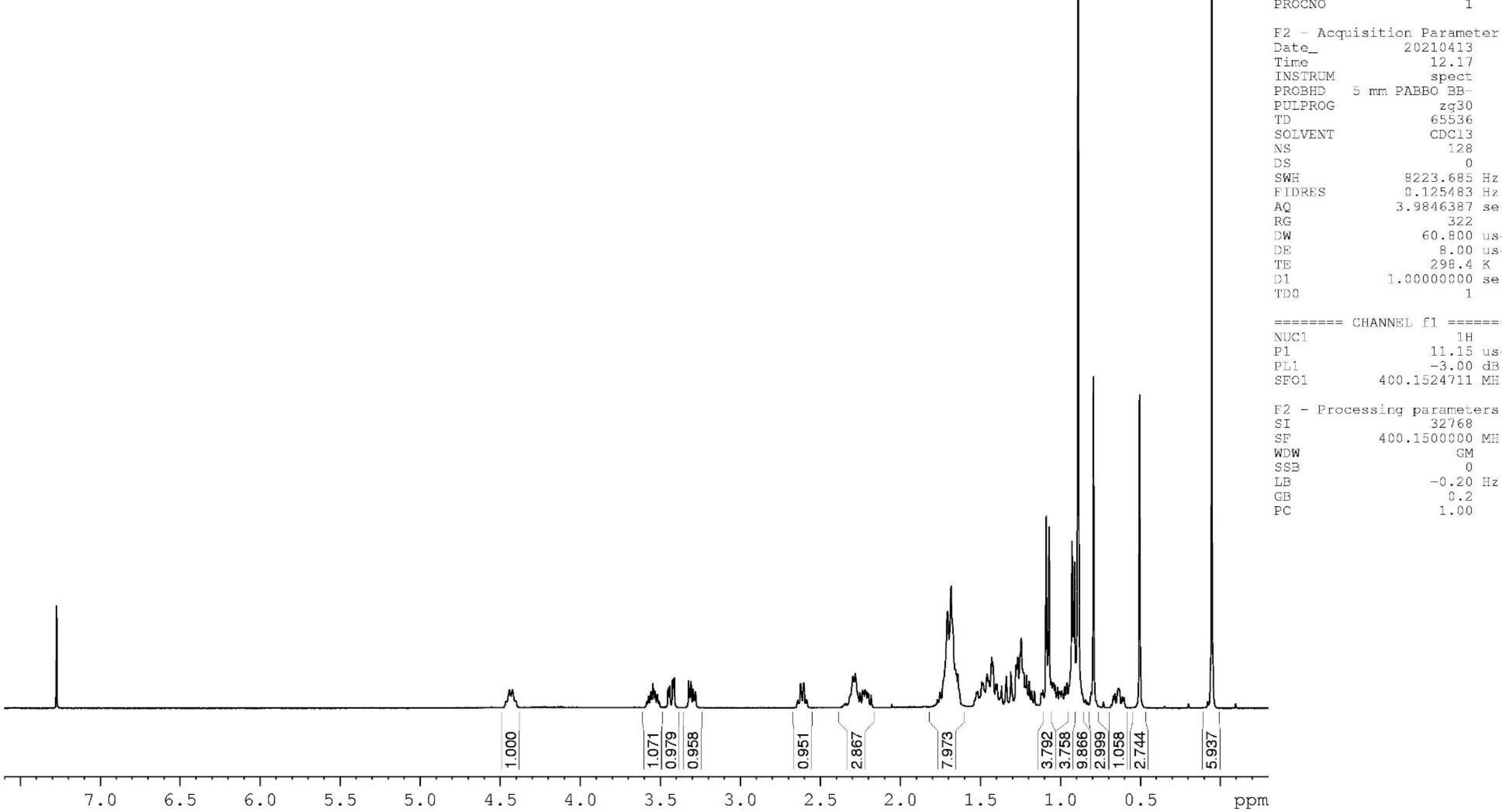

1.000
1.071
0.979
0.958
0.951
2.867
7.973
3.792
3.758
9.866
2.999
1.058
2.744
5.937
7.0
6.5
6.0
5.5
5.0
4.5
4.0
3.5
3.0
2.5
2.0
1.5
1.0
0.5
ppm
Current Data Parameters
NAME AW G344 (5)''(2)
EXPNO 1
PROCNO 1
F2 - Acquisition Parameter
Date_ 20210413
Time 12.17
INSTRUM spect
PROBHD 5 mm PABBO BB-
PULPROG zg30
TD 65536
SOLVENT CDCl3
NS 128
DS 0
SWH 8223.685 Hz
FIDRES 0.125483 Hz
AQ 3.9846387 se
RG 322
DW 60.800 us
DE 8.00 us
TE 298.4 K
D1 1.00000000 se
TD0 1
======== CHANNEL f1 ======
NUC1 1H
P1 11.15 us
PL1 -3.00 dB
SFO1 400.1524711 MH
F2 - Processing parameters
SI 32768
SF 400.1500000 MH
WDW GM
SSB 0
LB -0.20 Hz
GB 0.2
PC 1.00

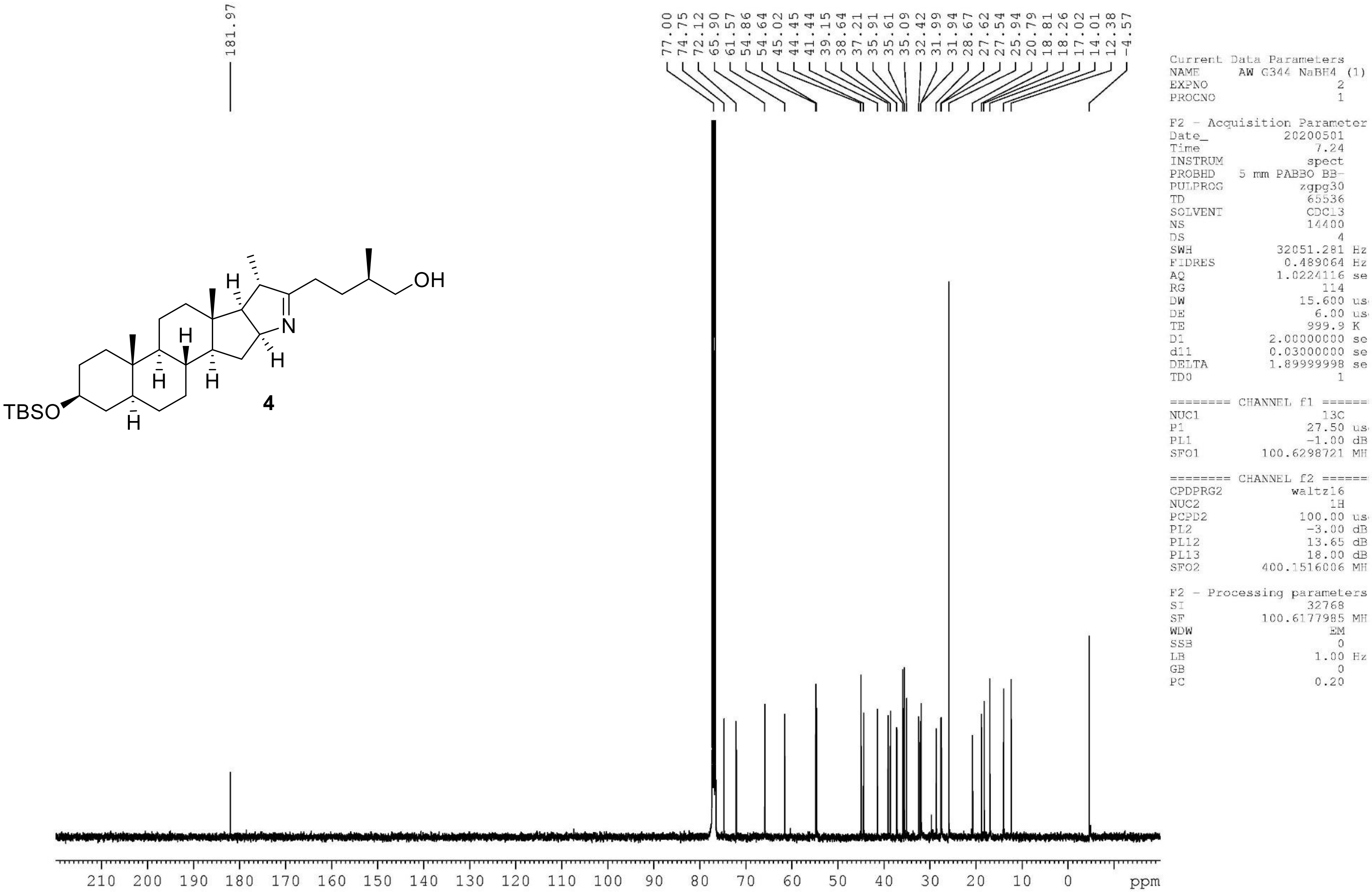
181.97
77.00
74.75
72.12
65.90
61.57
54.86
54.64
45.02
44.45
41.44
39.15
38.64
37.21
35.91
35.61
35.09
32.42
31.99
31.94
28.67
27.62
27.54
25.94
20.79
18.81
18.26
17.02
14.01
12.38
-4.57
TBSO
OH
N
H
4
Current Data Parameters
NAME AW G344 NaBH4 (1)
EXPNO 2
PROCNO 1
F2 - Acquisition Parameter
Date_ 20200501
Time 7.24
INSTRUM spect
PROBHD 5 mm PABBO BB-
PULPROG zgpg30
TD 65536
SOLVENT CDCl3
NS 14400
DS 4
SWH 32051.281 Hz
FIDRES 0.489064 Hz
AQ 1.0224116 se
RG 114
DW 15.600 us
DE 6.00 us
TE 999.9 K
D1 2.00000000 se
d11 0.03000000 se
DELTA 1.89999998 se
TD0 1
======== CHANNEL f1 ======
NUC1 13C
P1 27.50 us
PL1 -1.00 dB
SFO1 100.6298721 MH
======== CHANNEL f2 ======
CPDPRG2 waltz16
NUC2 1H
PCPD2 100.00 us
PL2 -3.00 dB
PL12 13.65 dB
PL13 18.00 dB
SFO2 400.1516006 MH
F2 - Processing parameters
SI 32768
SF 100.6177985 MH
WDW EM
SSB 0
LB 1.00 Hz
GB 0
PC 0.20
210 200 190 180 170 160 150 140 130 120 110 100 90 80 70 60 50 40 30 20 10 0 ppm

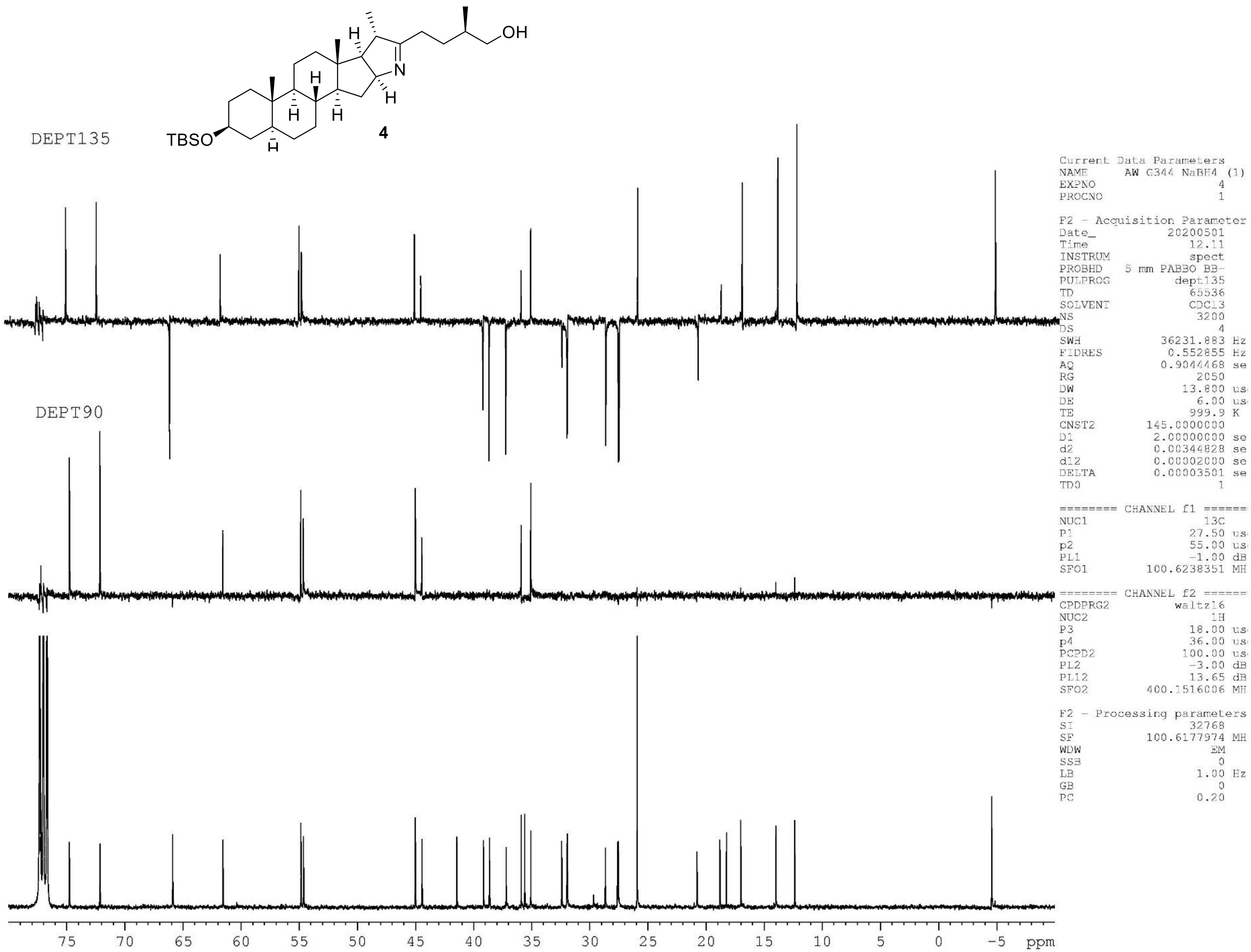

TBSO
OH
H
N
4
DEPT135
DEPT90
Current Data Parameters
NAME AW G344 NaBH4 (1)
EXPNO 4
PROCNO 1
F2 - Acquisition Parameter
Date_ 20200501
Time 12.11
INSTRUM spect
PROBHD 5 mm PABBO BB-
PULPROG dept135
TD 65536
SOLVENT CDCl3
NS 3200
DS 4
SWH 36231.883 Hz
FIDRES 0.552855 Hz
AQ 0.9044468 se
RG 2050
DW 13.800 us
DE 6.00 us
TE 999.9 K
CNST2 145.0000000
D1 2.00000000 se
d2 0.00344828 se
d12 0.00002000 se
DELTA 0.00003501 se
TD0 1
======== CHANNEL f1 ======
NUC1 13C
P1 27.50 us
p2 55.00 us
PL1 -1.00 dB
SFO1 100.6238351 MH
======== CHANNEL f2 ======
CPDPRG2 waltz16
NUC2 1H
P3 18.00 us
p4 36.00 us
PCPD2 100.00 us
PL2 -3.00 dB
PL12 13.65 dB
SFO2 400.1516006 MH
F2 - Processing parameters
SI 32768
SF 100.6177974 MH
WDW EM
SSB 0
LB 1.00 Hz
GB 0
PC 0.20
75 70 65 60 55 50 45 40 35 30 25 20 15 10 5 0 -5 ppm

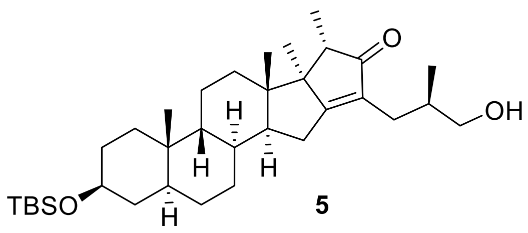
TBSO
OH
O
H

5

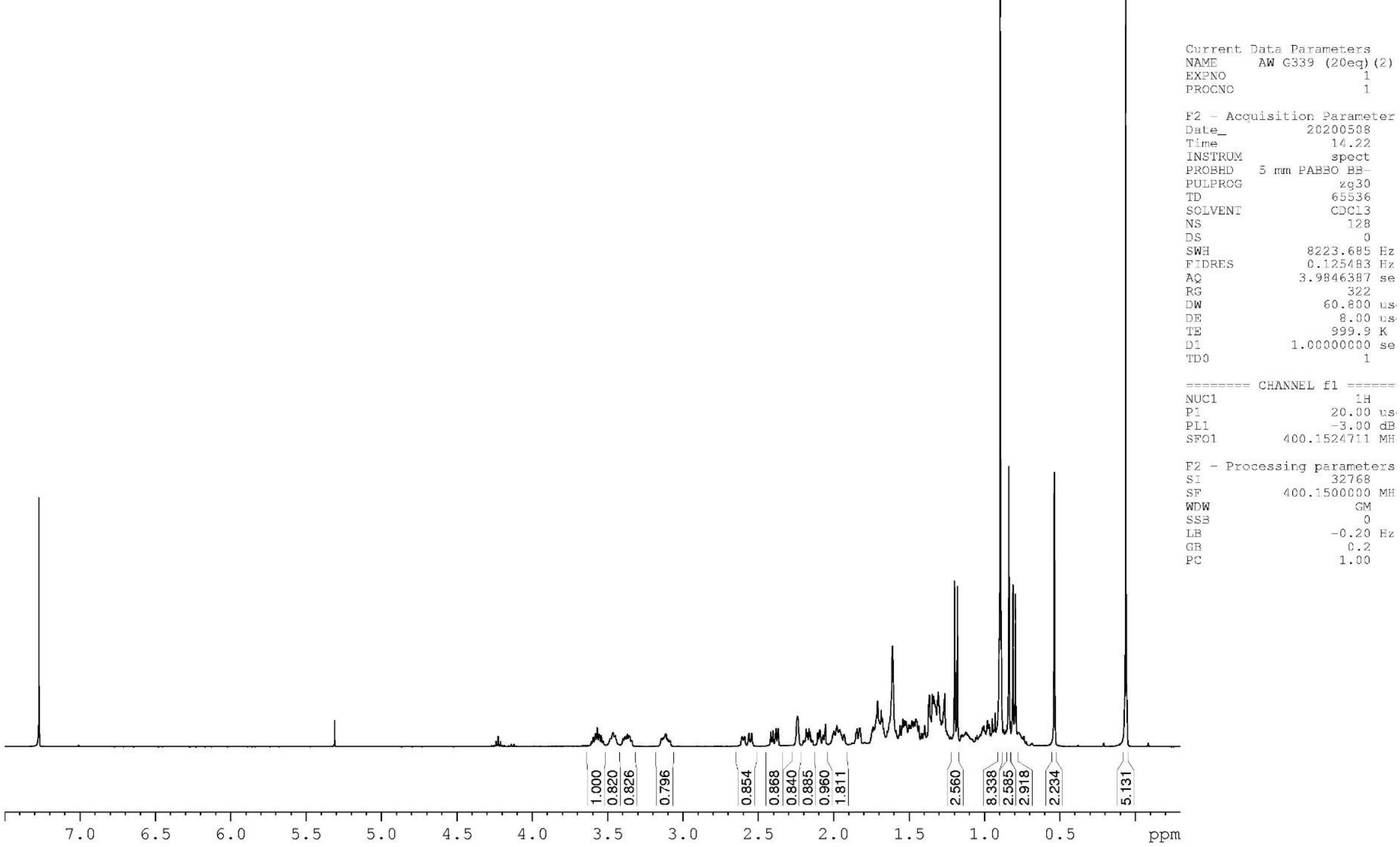
Current Data Parameters
NAME AW G339 (20eq)(2)
EXPNO 1
PROCNO 1
F2 - Acquisition Parameter
Date_ 20200508
Time 14.22
INSTRUM spect
PROBHD 5 mm PABBO BB-
PULPROG zg30
TD 65536
SOLVENT CDCl3
NS 128
DS 0
SWH 8223.685 Hz
FIDRES 0.125483 Hz
AQ 3.9846387 se
RG 322
DW 60.800 us
DE 8.00 us
TE 999.9 K
D1 1.00000000 se
TD0 1
======== CHANNEL f1 ======
NUC1 1H
P1 20.00 us
PL1 -3.00 dB
SFO1 400.1524711 MH
F2 - Processing parameters
SI 32768
SF 400.1500000 MH
WDW GM
SSB 0
LB -0.20 Hz
GB 0.2
PC 1.00
1.000
0.820
0.826
0.796
0.854
0.868
0.840
0.885
0.960
1.811
2.560
8.338
2.585
2.918
2.234
5.131
7.0
6.5
6.0
5.5
5.0
4.5
4.0
3.5
3.0
2.5
2.0
1.5
1.0
0.5
ppm

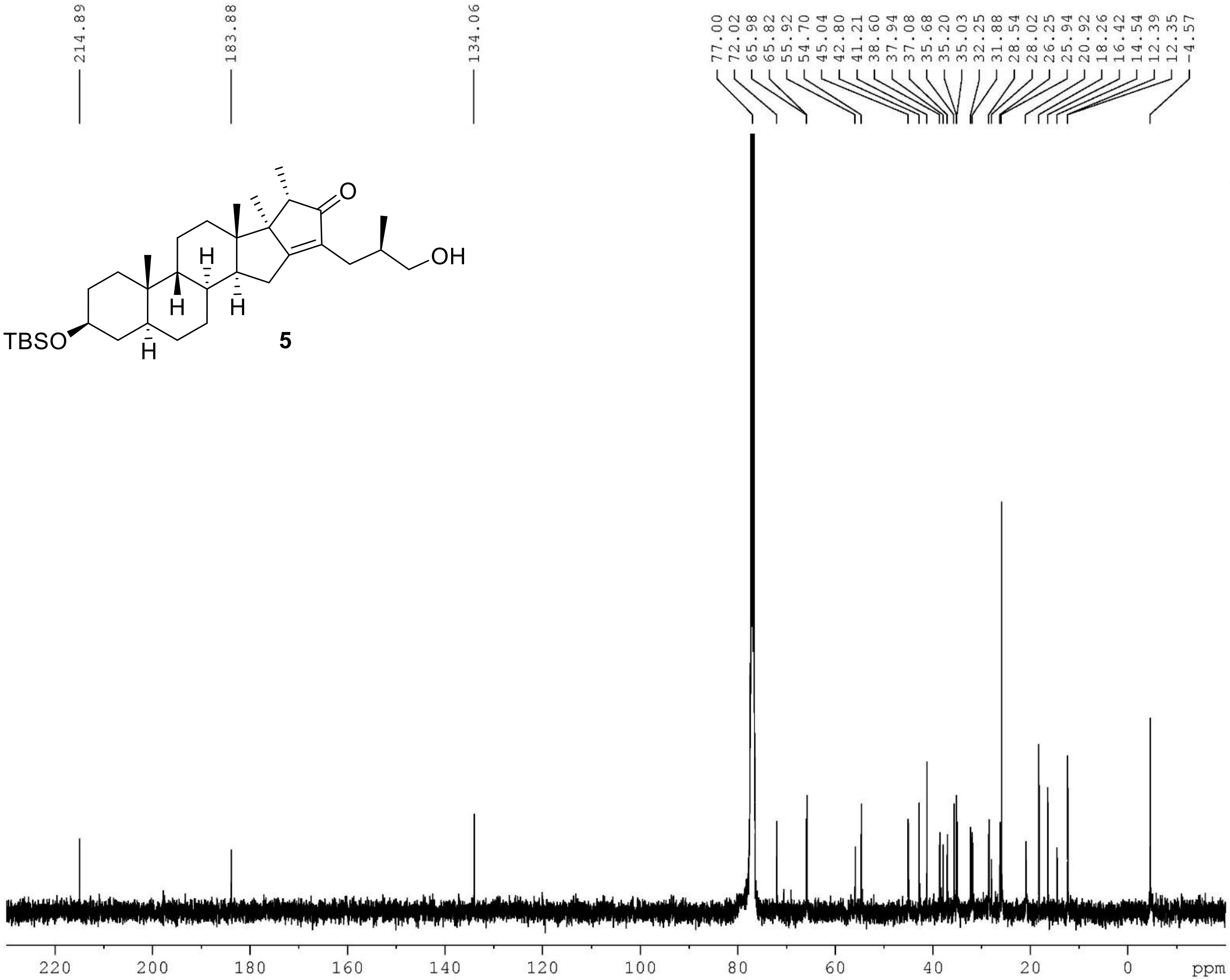


```
Current Data Parameters
NAME        UK RC23-P1F5
EXPNO                  2
PROCNO                 1

F2 - Acquisition Parameter
Date_           20190711
Time                5.56
INSTRUM            spect
PROBHD   5 mm PABBO BB-
PULPROG          zgpg30
TD                65536
SOLVENT            CDCl3
NS                12800
DS                    4
SWH           32051.281 Hz
FIDRES         0.489064 Hz
AQ            1.0224116 se
RG                  144
DW               15.600 us
DE                 6.00 us
TE                999.9 K
D1           2.00000000 se
d11          0.03000000 se
DELTA        1.89999998 se
TD0                   1

======== CHANNEL f1 ======
NUC1                13C
P1                27.50 us
PL1               -1.00 dB
SFO1        100.6298721 MH

======== CHANNEL f2 ======
CPDPRG2          waltz16
NUC2                 1H
PCPD2            100.00 us
PL2               -3.00 dB
PL12              13.65 dB
PL13              18.00 dB
SFO2        400.1516006 MH

F2 - Processing parameters
SI                32768
SF          100.6177989 MH
WDW                  EM
SSB                   0
LB                 1.00 Hz
GB                    0
PC                 0.20
```

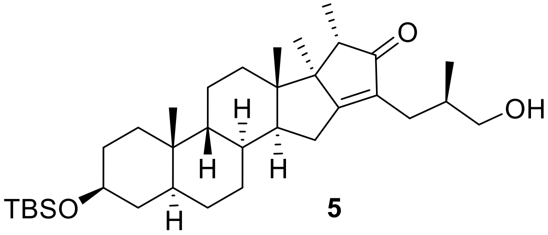
TBSO
OH
5

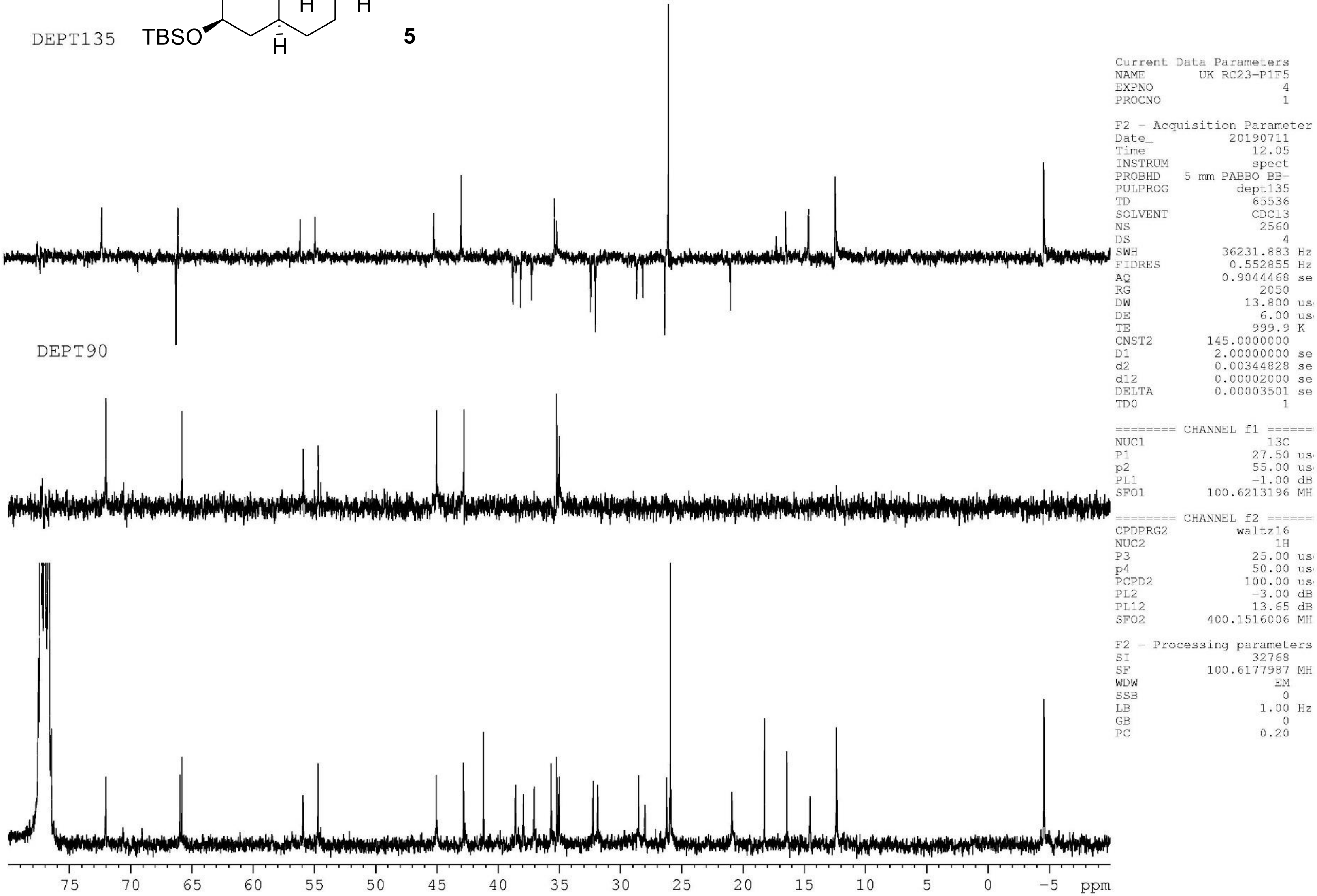
DEPT135
DEPT90
75 70 65 60 55 50 45 40 35 30 25 20 15 10 5 0 -5 ppm
Current Data Parameters
NAME UK RC23-P1F5
EXPNO 4
PROCNO 1
F2 - Acquisition Parameter
Date_ 20190711
Time 12.05
INSTRUM spect
PROBHD 5 mm PABBO BB-
PULPROG dept135
TD 65536
SOLVENT CDCl3
NS 2560
DS 4
SWH 36231.883 Hz
FIDRES 0.552855 Hz
AQ 0.9044468 se
RG 2050
DW 13.800 us
DE 6.00 us
TE 999.9 K
CNST2 145.0000000
D1 2.00000000 se
d2 0.00344828 se
d12 0.00002000 se
DELTA 0.00003501 se
TD0 1
======== CHANNEL f1 ======
NUC1 13C
P1 27.50 us
p2 55.00 us
PL1 -1.00 dB
SFO1 100.6213196 MH
======== CHANNEL f2 ======
CPDPRG2 waltz16
NUC2 1H
P3 25.00 us
p4 50.00 us
PCPD2 100.00 us
PL2 -3.00 dB
PL12 13.65 dB
SFO2 400.1516006 MH
F2 - Processing parameters
SI 32768
SF 100.6177987 MH
WDW EM
SSB 0
LB 1.00 Hz
GB 0
PC 0.20

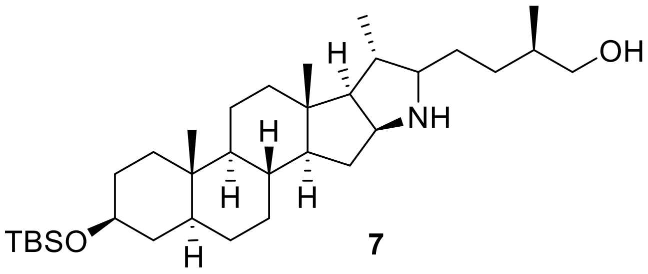
TBSO
OH
NH
H
H
H
H
7

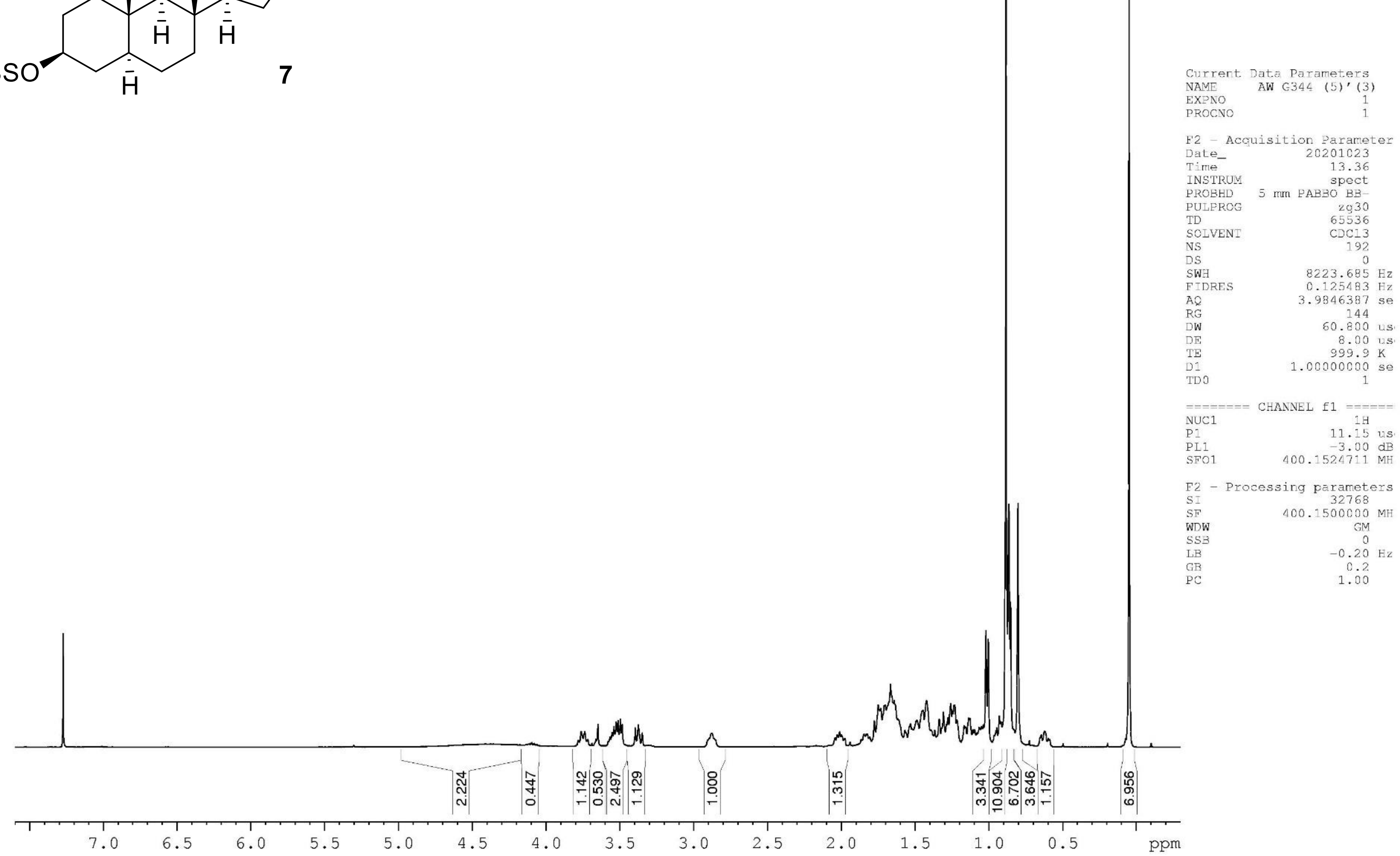
Current Data Parameters
NAME AW G344 (5)'(3)
EXPNO 1
PROCNO 1
F2 - Acquisition Parameter
Date_ 20201023
Time 13.36
INSTRUM spect
PROBHD 5 mm PABBO BB-
PULPROG zg30
TD 65536
SOLVENT CDCl3
NS 192
DS 0
SWH 8223.685 Hz
FIDRES 0.125483 Hz
AQ 3.9846387 se
RG 144
DW 60.800 us
DE 8.00 us
TE 999.9 K
D1 1.00000000 se
TD0 1
======== CHANNEL f1 ======
NUC1 1H
P1 11.15 us
PL1 -3.00 dB
SFO1 400.1524711 MH
F2 - Processing parameters
SI 32768
SF 400.1500000 MH
WDW GM
SSB 0
LB -0.20 Hz
GB 0.2
PC 1.00
2.224
0.447
1.142
0.530
2.497
1.129
1.000
1.315
3.341
10.904
6.702
3.646
1.157
6.956
7.0
6.5
6.0
5.5
5.0
4.5
4.0
3.5
3.0
2.5
2.0
1.5
1.0
0.5
ppm

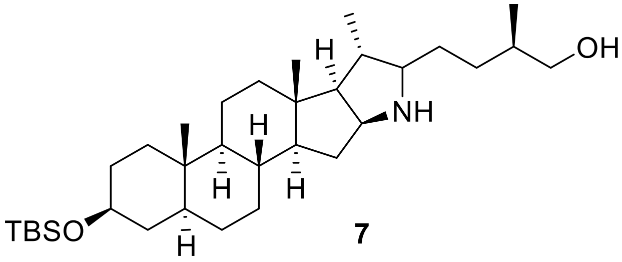

TBSO
H
H
H
H
H
NH
OH

7

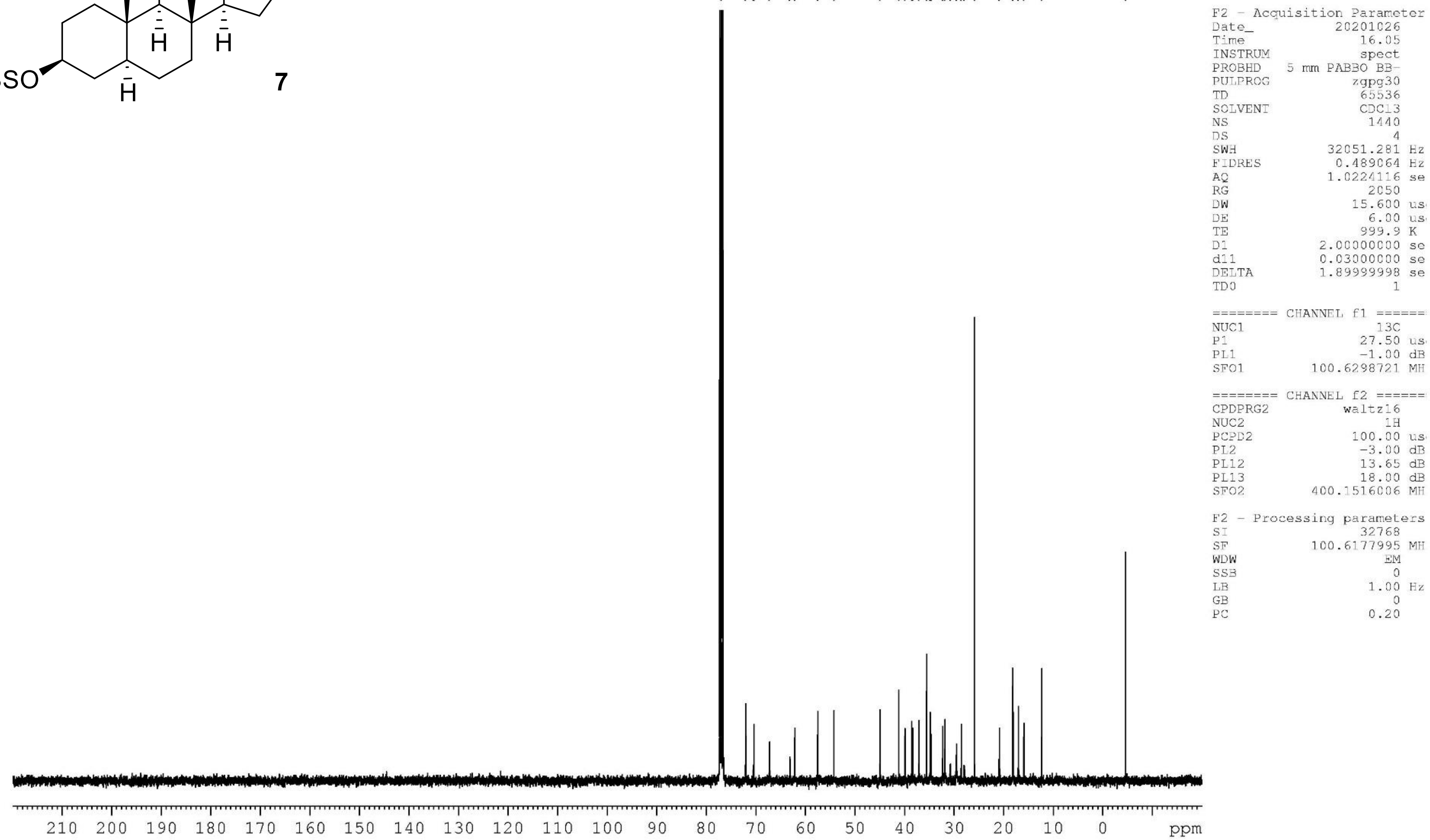

77.00
72.05
70.54
70.43
67.26
63.17
62.19
57.55
54.30
44.95
41.19
39.96
38.60
38.37
37.11
35.59
34.84
34.67
32.32
31.89
30.80
29.55
28.58
27.99
27.94
25.92
20.90
18.23
18.13
17.04
15.96
12.34
-4.58
Current Data Parameters
NAME AW G344 (5)'(3)
EXPNO 2
PROCNO 1
F2 - Acquisition Parameter
Date_ 20201026
Time 16.05
INSTRUM spect
PROBHD 5 mm PABBO BB-
PULPROG zgpg30
TD 65536
SOLVENT CDCl3
NS 1440
DS 4
SWH 32051.281 Hz
FIDRES 0.489064 Hz
AQ 1.0224116 se
RG 2050
DW 15.600 us
DE 6.00 us
TE 999.9 K
D1 2.00000000 se
d11 0.03000000 se
DELTA 1.89999998 se
TD0 1
======== CHANNEL f1 ======
NUC1 13C
P1 27.50 us
PL1 -1.00 dB
SFO1 100.6298721 MH
======== CHANNEL f2 ======
CPDPRG2 waltz16
NUC2 1H
PCPD2 100.00 us
PL2 -3.00 dB
PL12 13.65 dB
PL13 18.00 dB
SFO2 400.1516006 MH
F2 - Processing parameters
SI 32768
SF 100.6177995 MH
WDW EM
SSB 0
LB 1.00 Hz
GB 0
PC 0.20
210 200 190 180 170 160 150 140 130 120 110 100 90 80 70 60 50 40 30 20 10 0 ppm

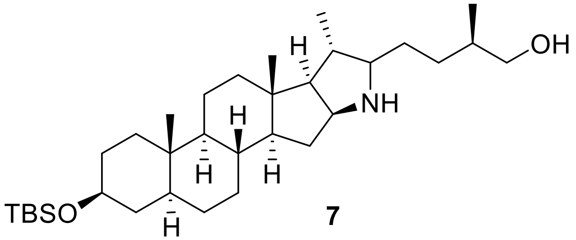

7

DEPT135

DEPT90

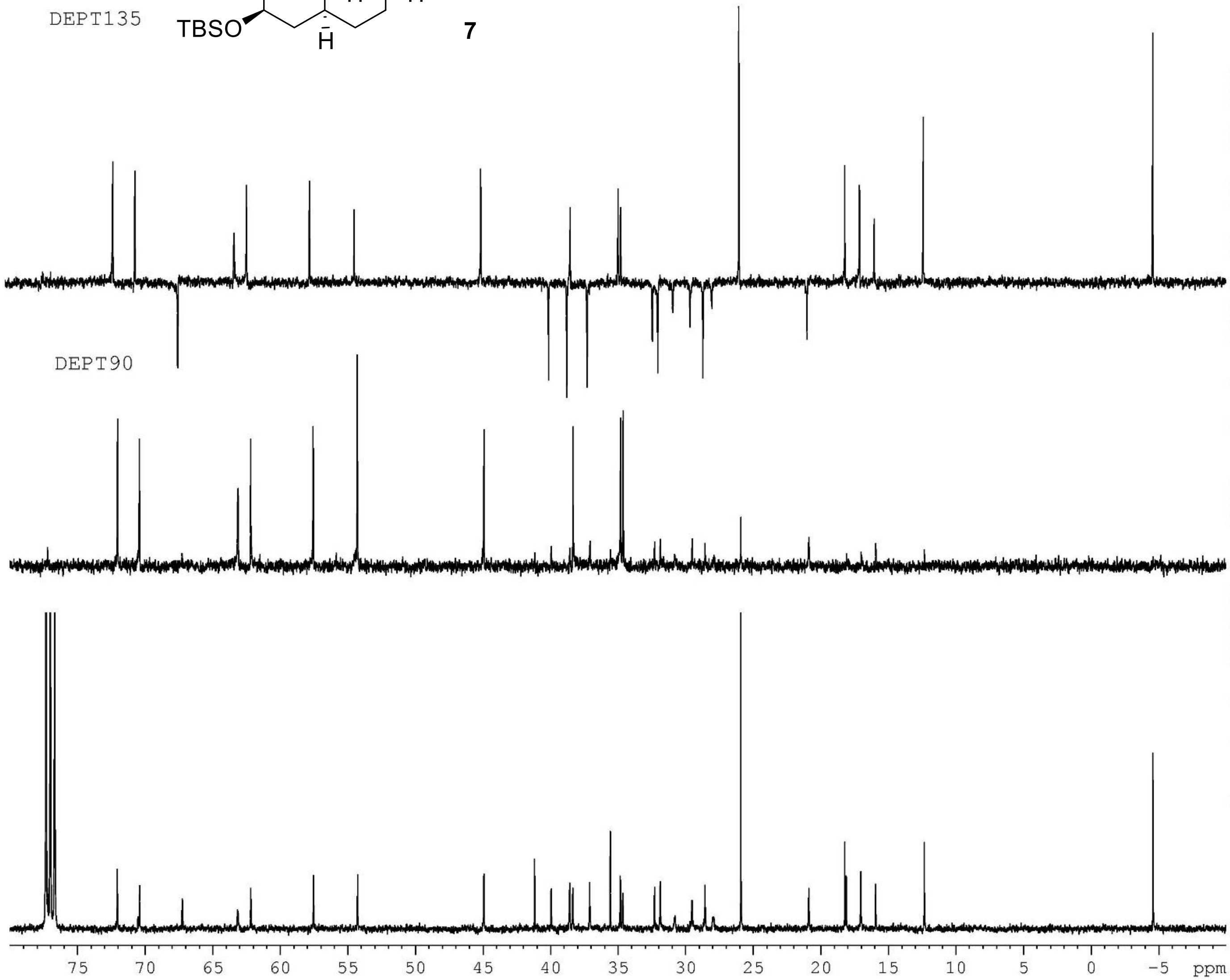


```
Current Data Parameters
NAME      AW G344 (5)'(3)
EXPNO                   4
PROCNO                  1

F2 - Acquisition Parameter
Date_            20201027
Time                16.32
INSTRUM             spect
PROBHD   5 mm PABBO BB-
PULPROG           dept135
TD                  65536
SOLVENT             CDCl3
NS                    512
DS                      4
SWH            36231.883 Hz
FIDRES          0.552855 Hz
AQ             0.9044468 se
RG                   2050
DW                 13.800 us
DE                   6.00 us
TE                  999.9 K
CNST2         145.0000000
D1             2.00000000 se
d2             0.00344828 se
d12            0.00002000 se
DELTA          0.00003501 se
TD0                     1

======== CHANNEL f1 ======
NUC1                  13C
P1                  27.50 us
p2                  55.00 us
PL1                 -1.00 dB
SFO1          100.6211184 MH

======== CHANNEL f2 ======
CPDPRG2           waltz16
NUC2                   1H
P3                  18.00 us
p4                  36.00 us
PCPD2              100.00 us
PL2                 -3.00 dB
PL12                13.65 dB
SFO2          400.1516006 MH

F2 - Processing parameters
SI                  32768
SF            100.6177996 MH
WDW                    EM
SSB                     0
LB                   1.00 Hz
GB                      0
PC                   1.40
```

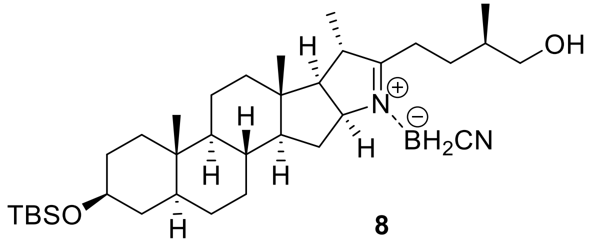

TBSO
H
H
H
H
H
H
N
BH2CN
OH

8

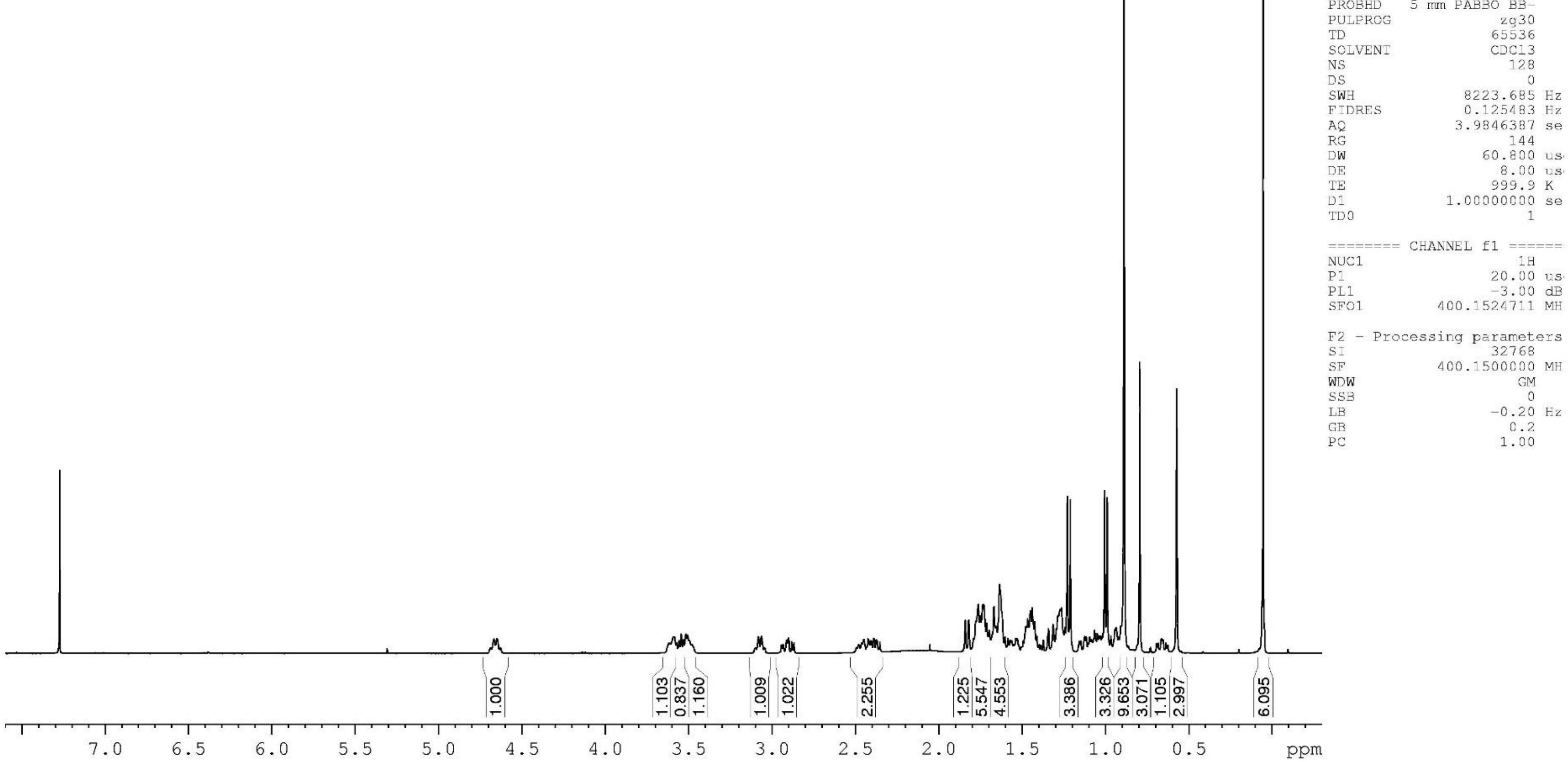

Current Data Parameters
NAME AW G344 (4)(1)
EXPNO 1
PROCNO 1
F2 - Acquisition Parameter
Date_ 20200511
Time 14.52
INSTRUM spect
PROBHD 5 mm PABBO BB-
PULPROG zg30
TD 65536
SOLVENT CDCl3
NS 128
DS 0
SWH 8223.685 Hz
FIDRES 0.125483 Hz
AQ 3.9846387 se
RG 144
DW 60.800 us
DE 8.00 us
TE 999.9 K
D1 1.00000000 se
TD0 1
======== CHANNEL f1 ======
NUC1 1H
P1 20.00 us
PL1 -3.00 dB
SFO1 400.1524711 MH
F2 - Processing parameters
SI 32768
SF 400.1500000 MH
WDW GM
SSB 0
LB -0.20 Hz
GB 0.2
PC 1.00
1.000
1.103
0.837
1.160
1.009
1.022
2.255
1.225
5.547
4.553
3.386
3.326
9.653
3.071
1.105
2.997
6.095
7.0
6.5
6.0
5.5
5.0
4.5
4.0
3.5
3.0
2.5
2.0
1.5
1.0
0.5
ppm

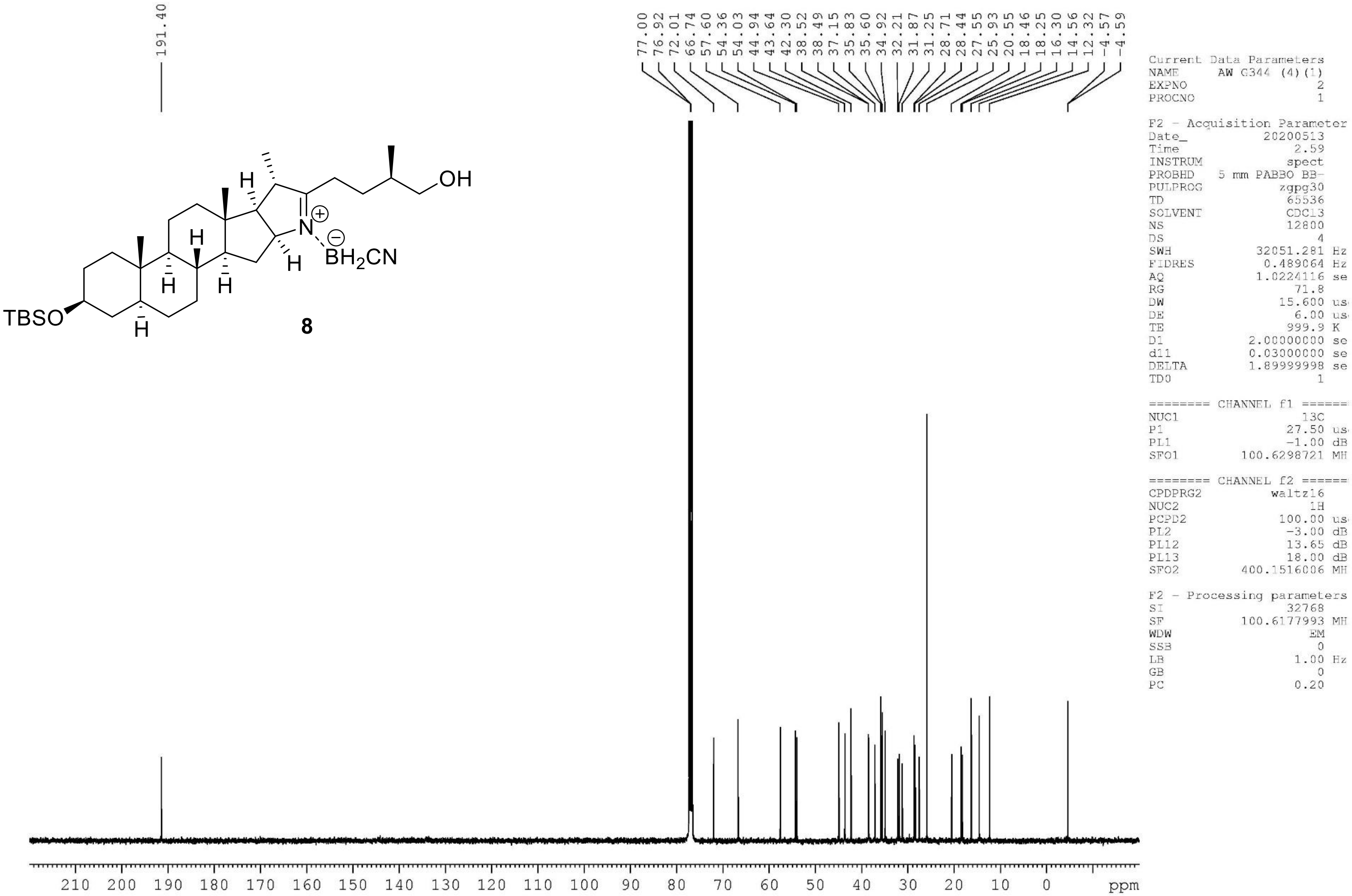
OH
H
N
BH2CN
H
H
H
H
TBSO
H
191.40
77.00
76.92
72.01
66.74
57.60
54.36
54.03
44.94
43.64
42.30
38.52
38.49
37.15
35.83
35.60
34.92
32.21
31.87
31.25
28.71
28.44
27.55
25.93
20.55
18.46
18.25
16.30
14.56
12.32
-4.57
-4.59
Current Data Parameters
NAME AW G344 (4)(1)
EXPNO 2
PROCNO 1
F2 - Acquisition Parameter
Date_ 20200513
Time 2.59
INSTRUM spect
PROBHD 5 mm PABBO BB-
PULPROG zgpg30
TD 65536
SOLVENT CDCl3
NS 12800
DS 4
SWH 32051.281 Hz
FIDRES 0.489064 Hz
AQ 1.0224116 se
RG 71.8
DW 15.600 us
DE 6.00 us
TE 999.9 K
D1 2.00000000 se
d11 0.03000000 se
DELTA 1.89999998 se
TD0 1
======== CHANNEL f1 ======
NUC1 13C
P1 27.50 us
PL1 -1.00 dB
SFO1 100.6298721 MH
======== CHANNEL f2 ======
CPDPRG2 waltz16
NUC2 1H
PCPD2 100.00 us
PL2 -3.00 dB
PL12 13.65 dB
PL13 18.00 dB
SFO2 400.1516006 MH
F2 - Processing parameters
SI 32768
SF 100.6177993 MH
WDW EM
SSB 0
LB 1.00 Hz
GB 0
PC 0.20
210 200 190 180 170 160 150 140 130 120 110 100 90 80 70 60 50 40 30 20 10 0 ppm

8

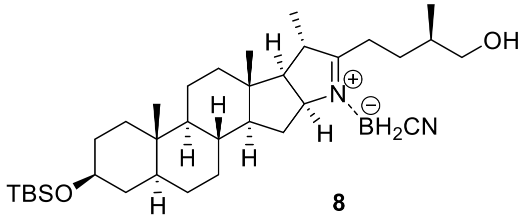

8

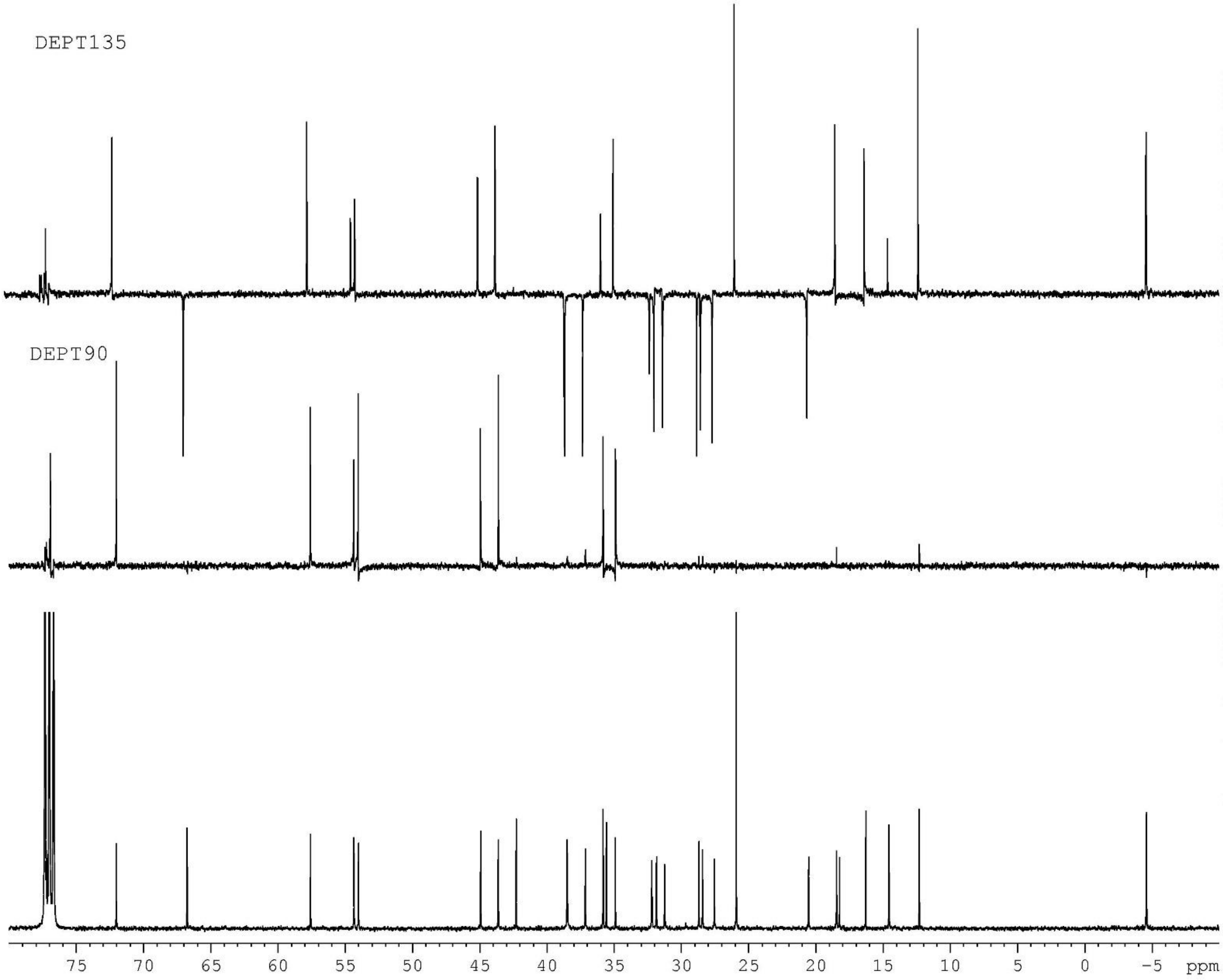


```
Current Data Parameters
NAME        AW G344 (4)(1)
EXPNO                    4
PROCNO                   1

F2 - Acquisition Parameter
Date_             20200513
Time                  9.02
INSTRUM              spect
PROBHD   5 mm PABBO BB-
PULPROG            dept135
TD                   65536
SOLVENT              CDCl3
NS                    4096
DS                       4
SWH              36231.883 Hz
FIDRES            0.552855 Hz
AQ               0.9044468 se
RG                    2050
DW                  13.800 us
DE                    6.00 us
TE                   999.9 K
CNST2          145.0000000
D1              2.00000000 se
d2              0.00344828 se
d12             0.00002000 se
DELTA           0.00003501 se
TD0                      1

======== CHANNEL f1 ======
NUC1                   13C
P1                   27.50 us
p2                   55.00 us
PL1                  -1.00 dB
SFO1           100.6228289 MH

======== CHANNEL f2 ======
CPDPRG2            waltz16
NUC2                    1H
P3                   18.00 us
p4                   36.00 us
PCPD2               100.00 us
PL2                  -3.00 dB
PL12                 13.65 dB
SFO2           400.1516006 MH

F2 - Processing parameters
SI                   32768
SF             100.6177990 MH
WDW                     EM
SSB                      0
LB                    1.00 Hz
GB                       0
PC                    0.20
```

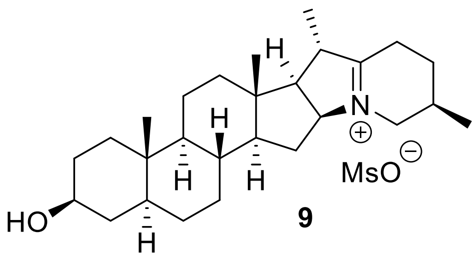

HO
H
H
H
H
H
N
MsO

9

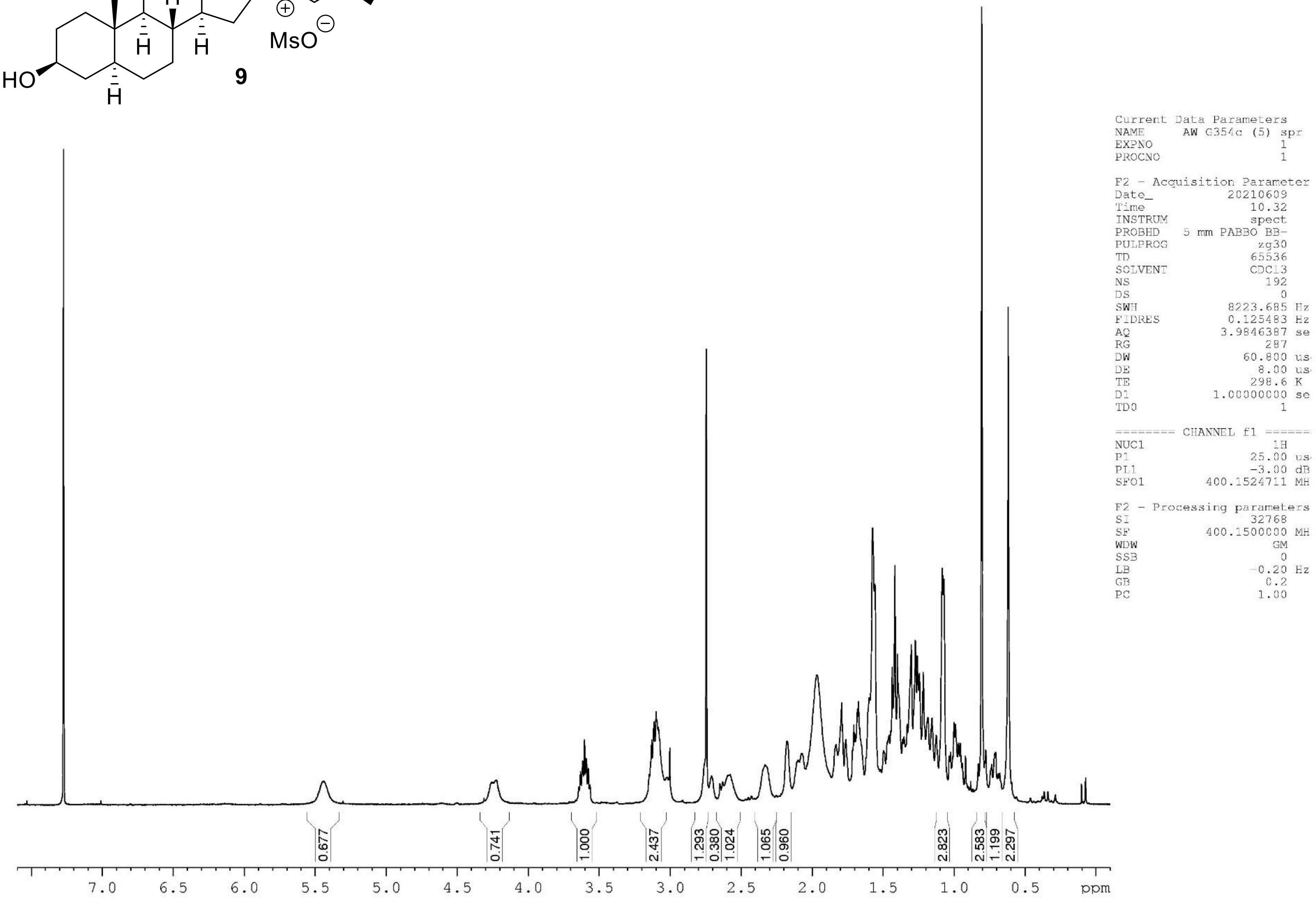

0.677
0.741
1.000
2.437
1.293
0.380
1.024
1.065
0.960
2.823
2.583
1.199
2.297
7.0 6.5 6.0 5.5 5.0 4.5 4.0 3.5 3.0 2.5 2.0 1.5 1.0 0.5 ppm
Current Data Parameters
NAME AW G354c (5) spr
EXPNO 1
PROCNO 1
F2 - Acquisition Parameter
Date_ 20210609
Time 10.32
INSTRUM spect
PROBHD 5 mm PABBO BB-
PULPROG zg30
TD 65536
SOLVENT CDCl3
NS 192
DS 0
SWH 8223.685 Hz
FIDRES 0.125483 Hz
AQ 3.9846387 se
RG 287
DW 60.800 us
DE 8.00 us
TE 298.6 K
D1 1.00000000 se
TD0 1
======== CHANNEL f1 ======
NUC1 1H
P1 25.00 us
PL1 -3.00 dB
SFO1 400.1524711 MH
F2 - Processing parameters
SI 32768
SF 400.1500000 MH
WDW GM
SSB 0
LB -0.20 Hz
GB 0.2
PC 1.00

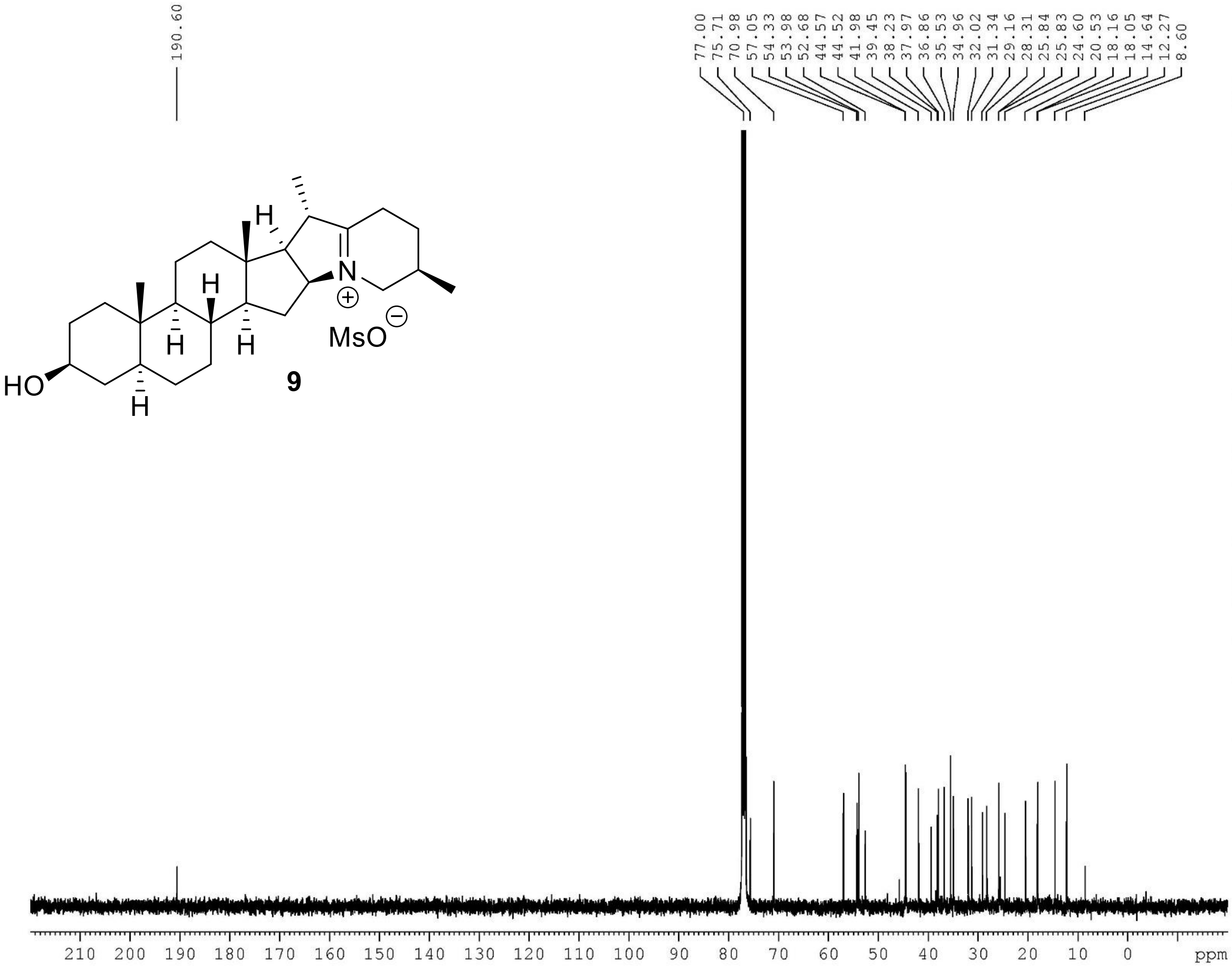


```
Current Data Parameters
NAME      AW G354c (5) susz
EXPNO                2
PROCNO               1

F2 - Acquisition Parameter
Date_          20210417
Time               7.37
INSTRUM           spect
PROBHD   5 mm PABBO BB-
PULPROG         zgpg30
TD               65536
SOLVENT          CDCl3
NS               16384
DS                   4
SWH          32051.281 Hz
FIDRES        0.489064 Hz
AQ           1.0224116 se
RG                 114
DW              15.600 us
DE                6.00 us
TE               299.0 K
D1          2.00000000 se
d11         0.03000000 se
DELTA       1.89999998 se
TD0                  1

======== CHANNEL f1 ======
NUC1               13C
P1               27.50 us
PL1              -1.00 dB
SFO1       100.6298721 MH

======== CHANNEL f2 ======
CPDPRG2         waltz16
NUC2                 1H
PCPD2           100.00 us
PL2              -3.00 dB
PL12             13.65 dB
PL13             18.00 dB
SFO2       400.1516006 MH

F2 - Processing parameters
SI               32768
SF         100.6177990 MH
WDW                 EM
SSB                  0
LB                1.00 Hz
GB                   0
PC                0.20
```

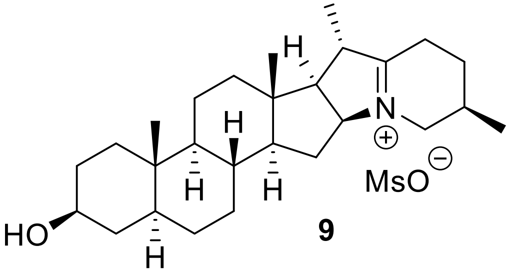
HO
H
N
MsO

9

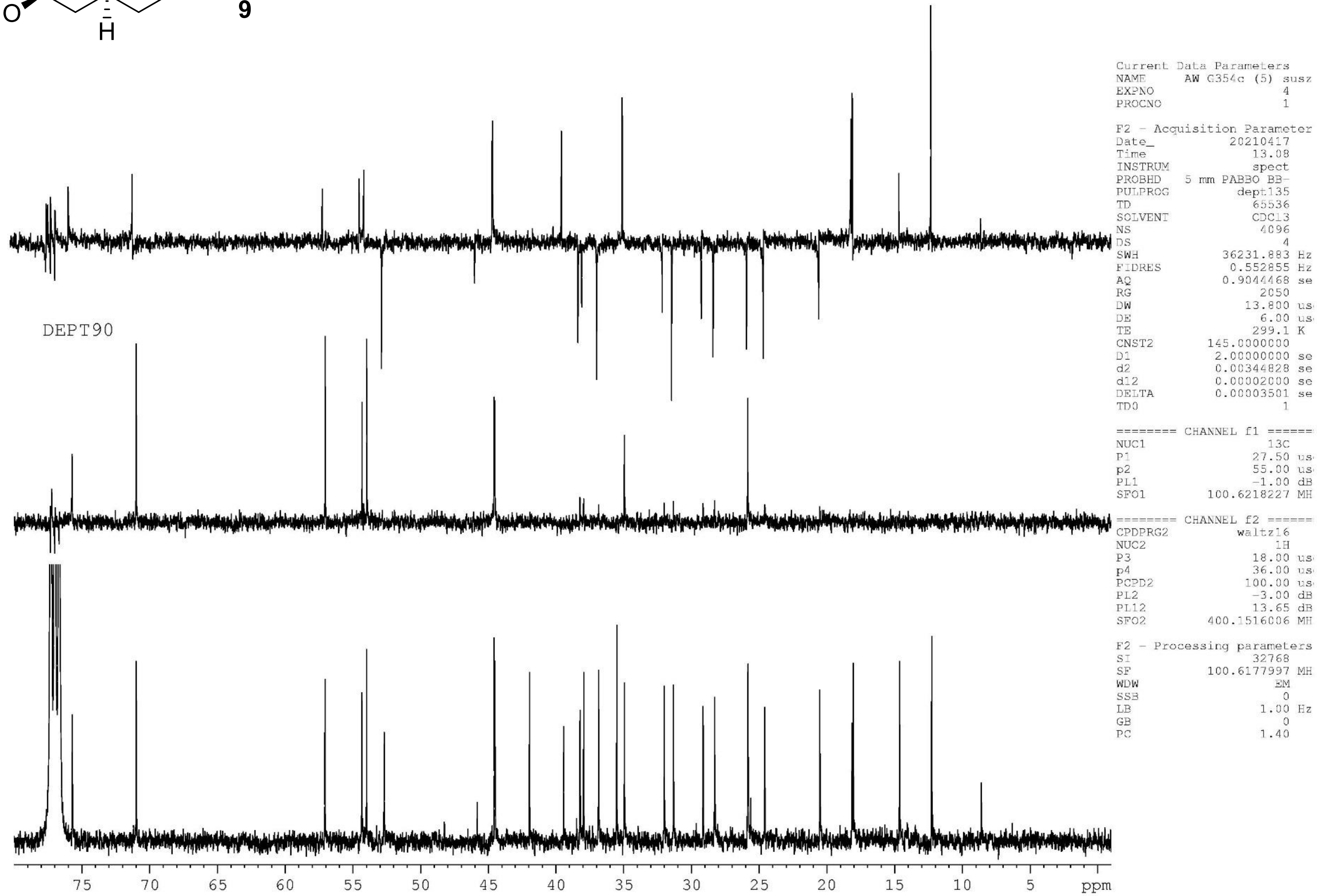
DEPT90
75
70
65
60
55
50
45
40
35
30
25
20
15
10
5
ppm
Current Data Parameters
NAME AW G354c (5) susz
EXPNO 4
PROCNO 1
F2 - Acquisition Parameter
Date_ 20210417
Time 13.08
INSTRUM spect
PROBHD 5 mm PABBO BB-
PULPROG dept135
TD 65536
SOLVENT CDCl3
NS 4096
DS 4
SWH 36231.883 Hz
FIDRES 0.552855 Hz
AQ 0.9044468 se
RG 2050
DW 13.800 us
DE 6.00 us
TE 299.1 K
CNST2 145.0000000
D1 2.00000000 se
d2 0.00344828 se
d12 0.00002000 se
DELTA 0.00003501 se
TD0 1
======== CHANNEL f1 ======
NUC1 13C
P1 27.50 us
p2 55.00 us
PL1 -1.00 dB
SFO1 100.6218227 MH
======== CHANNEL f2 ======
CPDPRG2 waltz16
NUC2 1H
P3 18.00 us
p4 36.00 us
PCPD2 100.00 us
PL2 -3.00 dB
PL12 13.65 dB
SFO2 400.1516006 MH
F2 - Processing parameters
SI 32768
SF 100.6177997 MH
WDW EM
SSB 0
LB 1.00 Hz
GB 0
PC 1.40

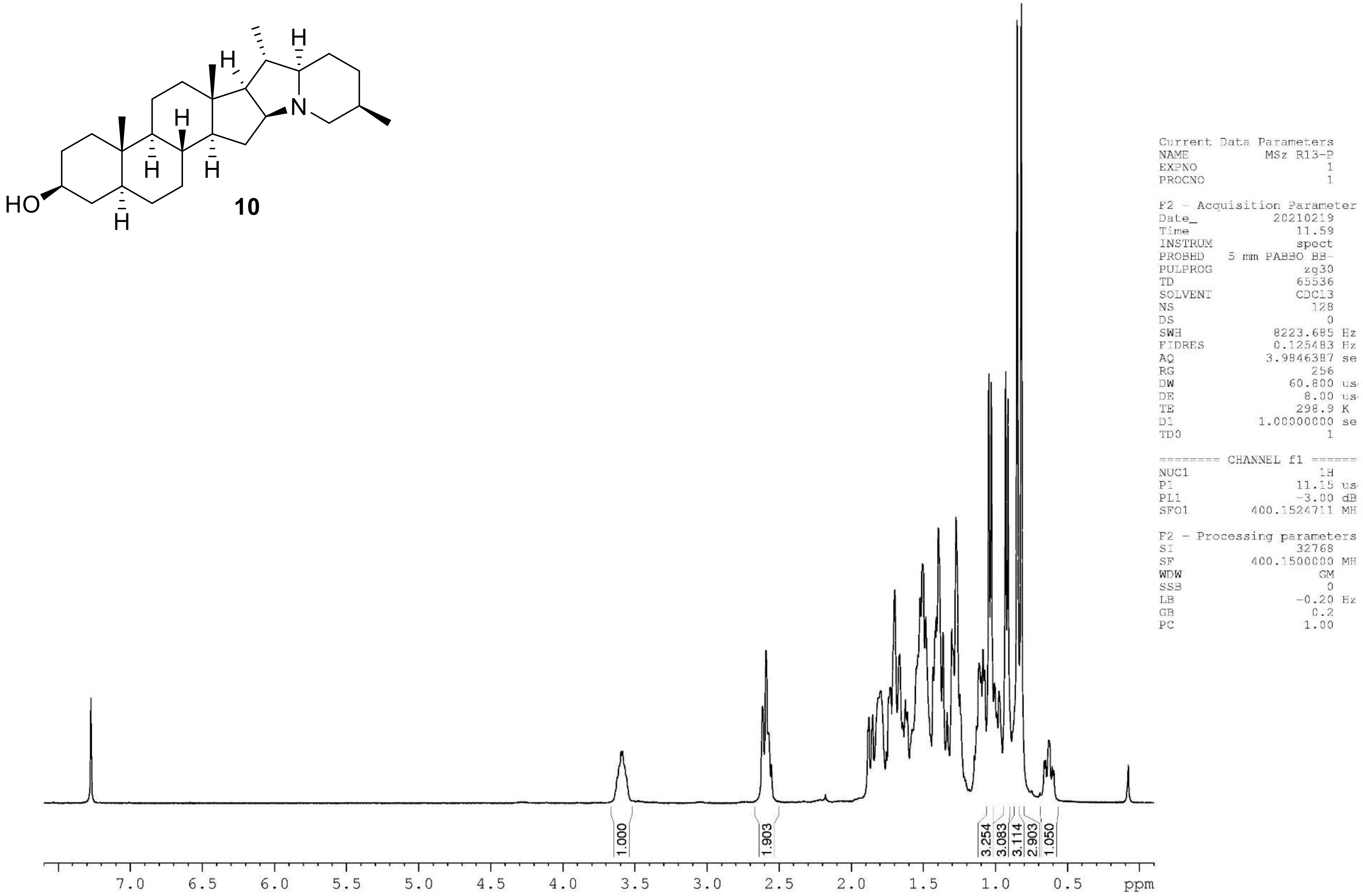
10
Current Data Parameters
NAME MSz R13-P
EXPNO 1
PROCNO 1
F2 - Acquisition Parameter
Date_ 20210219
Time 11.59
INSTRUM spect
PROBHD 5 mm PABBO BB-
PULPROG zg30
TD 65536
SOLVENT CDCl3
NS 128
DS 0
SWH 8223.685 Hz
FIDRES 0.125483 Hz
AQ 3.9846387 se
RG 256
DW 60.800 us
DE 8.00 us
TE 298.9 K
D1 1.00000000 se
TD0 1
======== CHANNEL f1 ======
NUC1 1H
P1 11.15 us
PL1 -3.00 dB
SFO1 400.1524711 MH
F2 - Processing parameters
SI 32768
SF 400.1500000 MH
WDW GM
SSB 0
LB -0.20 Hz
GB 0.2
PC 1.00
1.000
1.903
3.254
3.083
3.114
2.903
1.050
7.0
6.5
6.0
5.5
5.0
4.5
4.0
3.5
3.0
2.5
2.0
1.5
1.0
0.5
ppm

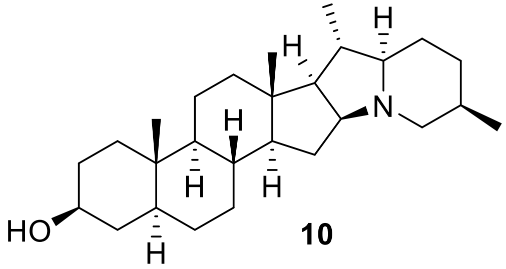

10

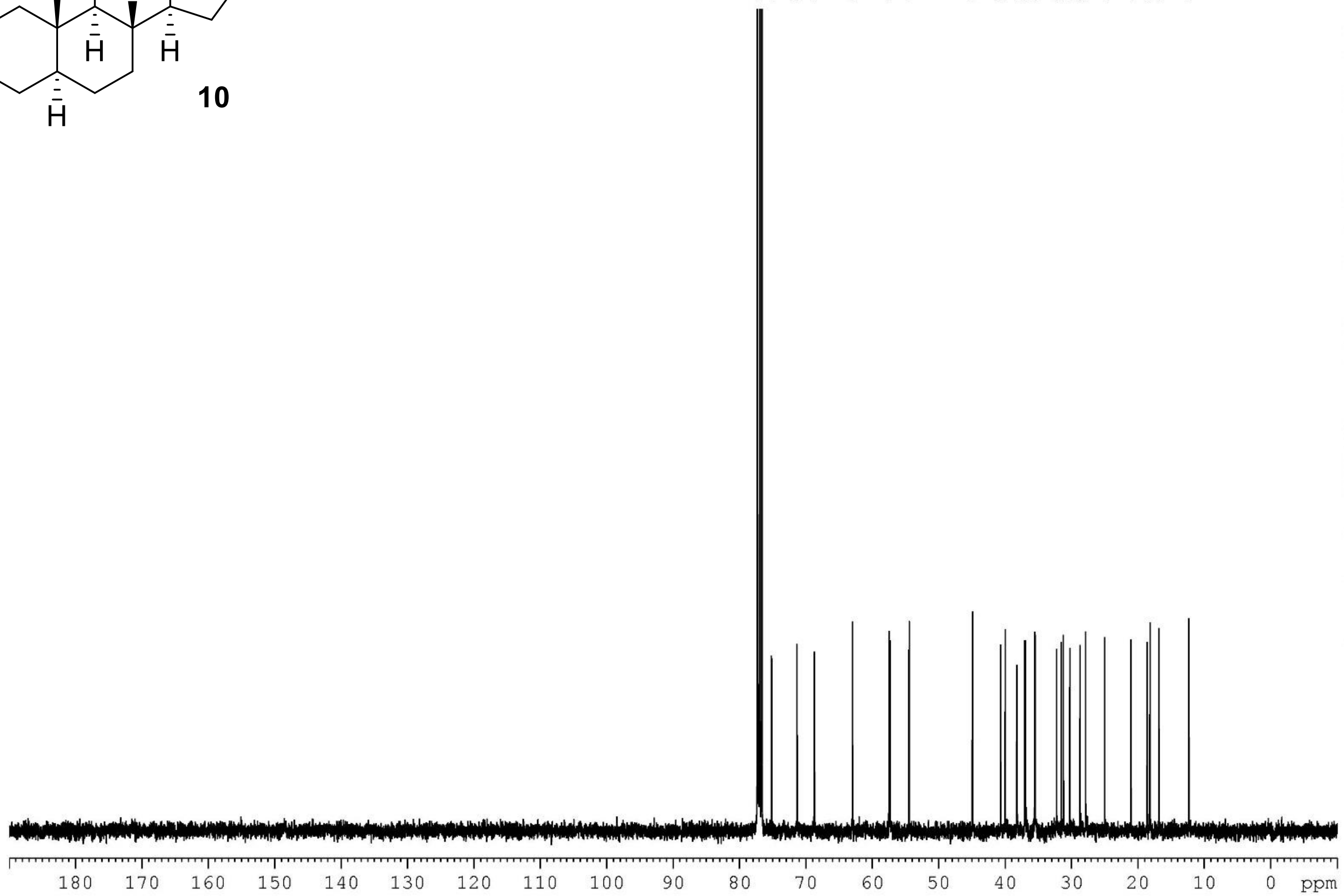

Current Data Parameters

NAME	MSz R13-P
EXPNO	2
PROCNO	1

F2 - Acquisition Parameter

Date_	20210219
Time	12.07
INSTRUM	spect
PROBHD	5 mm PABBO BB-
PULPROG	zgpg30
TD	65536
SOLVENT	CDCl3
NS	1920
DS	4
SWH	32051.281 Hz
FIDRES	0.489064 Hz
AQ	1.0224116 se
RG	2050
DW	15.600 us
DE	6.00 us
TE	299.2 K
D1	2.00000000 se
d11	0.03000000 se
DELTA	1.89999998 se
TD0	1

======== CHANNEL f1 ======

NUC1	13C
P1	27.50 us
PL1	-1.00 dB
SFO1	100.6298721 MH

======== CHANNEL f2 ======

CPDPRG2	waltz16
NUC2	1H
PCPD2	100.00 us
PL2	-3.00 dB
PL12	13.65 dB
PL13	18.00 dB
SFO2	400.1516006 MH

F2 - Processing parameters

SI	32768
SF	100.6177973 MH
WDW	EM
SSB	0
LB	1.00 Hz
GB	0
PC	0.20

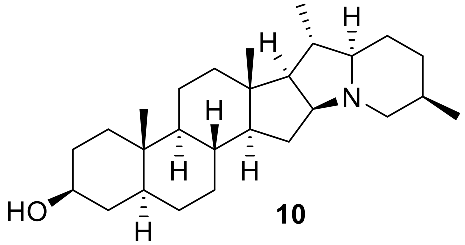
HO
H
H
H
H
H
N

10

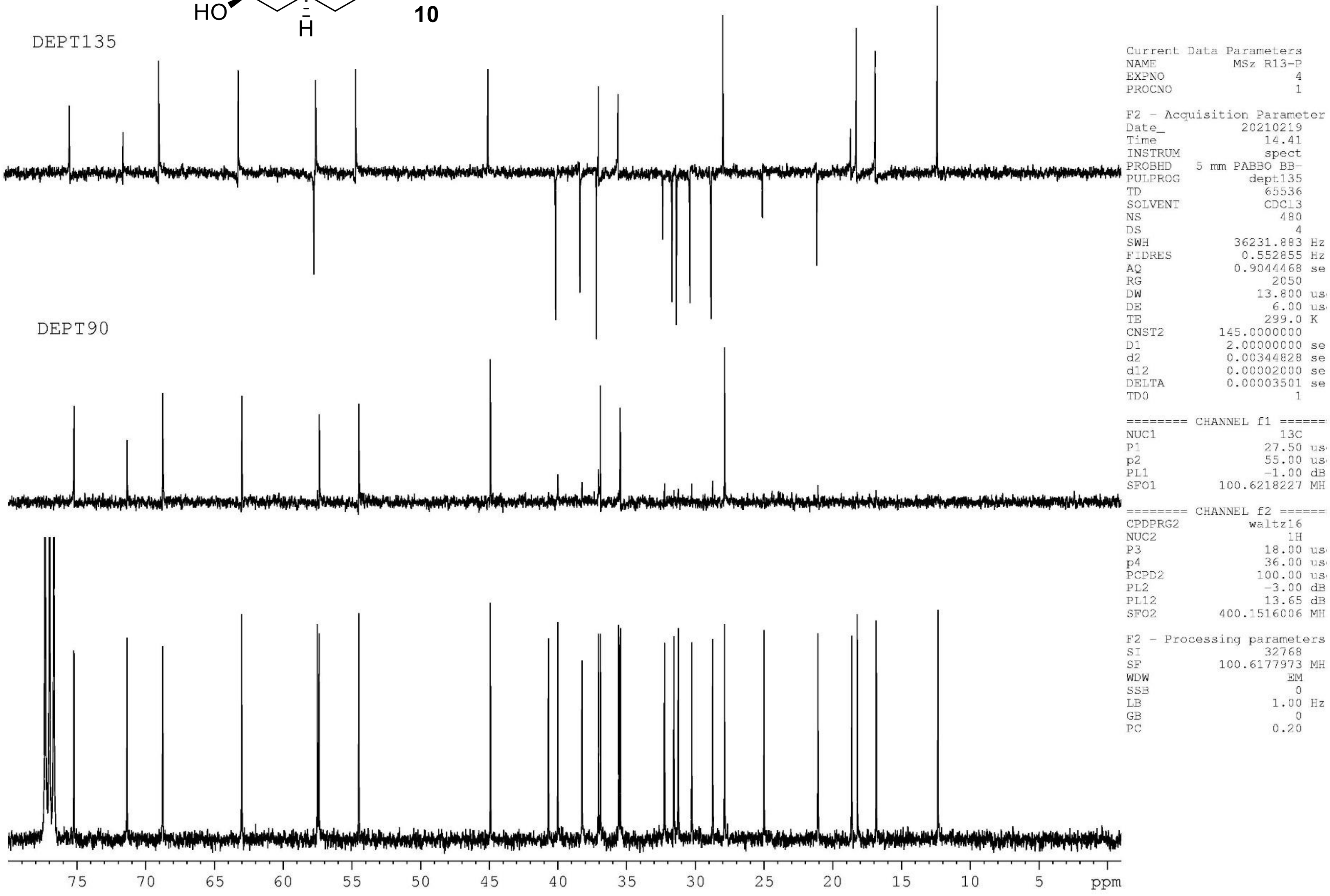
DEPT135
DEPT90
75
70
65
60
55
50
45
40
35
30
25
20
15
10
5
ppm
Current Data Parameters
NAME MSz R13-P
EXPNO 4
PROCNO 1
F2 - Acquisition Parameter
Date_ 20210219
Time 14.41
INSTRUM spect
PROBHD 5 mm PABBO BB-
PULPROG dept135
TD 65536
SOLVENT CDCl3
NS 480
DS 4
SWH 36231.883 Hz
FIDRES 0.552855 Hz
AQ 0.9044468 se
RG 2050
DW 13.800 us
DE 6.00 us
TE 299.0 K
CNST2 145.0000000
D1 2.00000000 se
d2 0.00344828 se
d12 0.00002000 se
DELTA 0.00003501 se
TD0 1
======== CHANNEL f1 ======
NUC1 13C
P1 27.50 us
p2 55.00 us
PL1 -1.00 dB
SFO1 100.6218227 MH
======== CHANNEL f2 ======
CPDPRG2 waltz16
NUC2 1H
P3 18.00 us
p4 36.00 us
PCPD2 100.00 us
PL2 -3.00 dB
PL12 13.65 dB
SFO2 400.1516006 MH
F2 - Processing parameters
SI 32768
SF 100.6177973 MH
WDW EM
SSB 0
LB 1.00 Hz
GB 0
PC 0.20

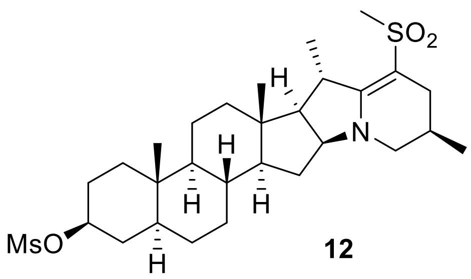

SO2
H
H
H
H
N
MsO
H

12

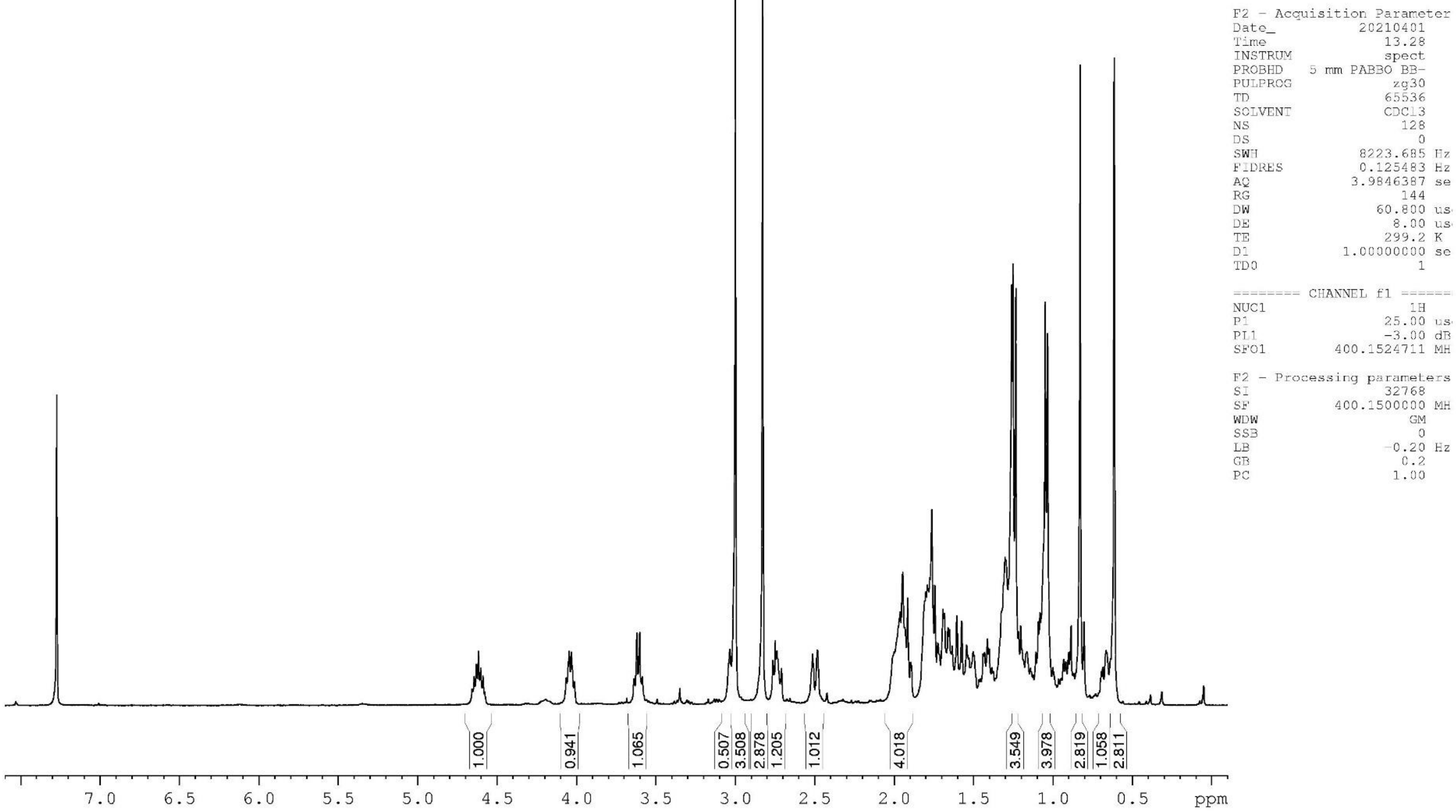

1.000
0.941
1.065
0.507
3.508
2.878
1.205
1.012
4.018
3.549
3.978
2.819
1.058
2.811
7.0
6.5
6.0
5.5
5.0
4.5
4.0
3.5
3.0
2.5
2.0
1.5
1.0
0.5
ppm
Current Data Parameters
NAME AW G354b II (1)
EXPNO 1
PROCNO 1
F2 - Acquisition Parameter
Date_ 20210401
Time 13.28
INSTRUM spect
PROBHD 5 mm PABBO BB-
PULPROG zg30
TD 65536
SOLVENT CDCl3
NS 128
DS 0
SWH 8223.685 Hz
FIDRES 0.125483 Hz
AQ 3.9846387 se
RG 144
DW 60.800 us
DE 8.00 us
TE 299.2 K
D1 1.00000000 se
TD0 1
======== CHANNEL f1 ======
NUC1 1H
P1 25.00 us
PL1 -3.00 dB
SFO1 400.1524711 MH
F2 - Processing parameters
SI 32768
SF 400.1500000 MH
WDW GM
SSB 0
LB -0.20 Hz
GB 0.2
PC 1.00

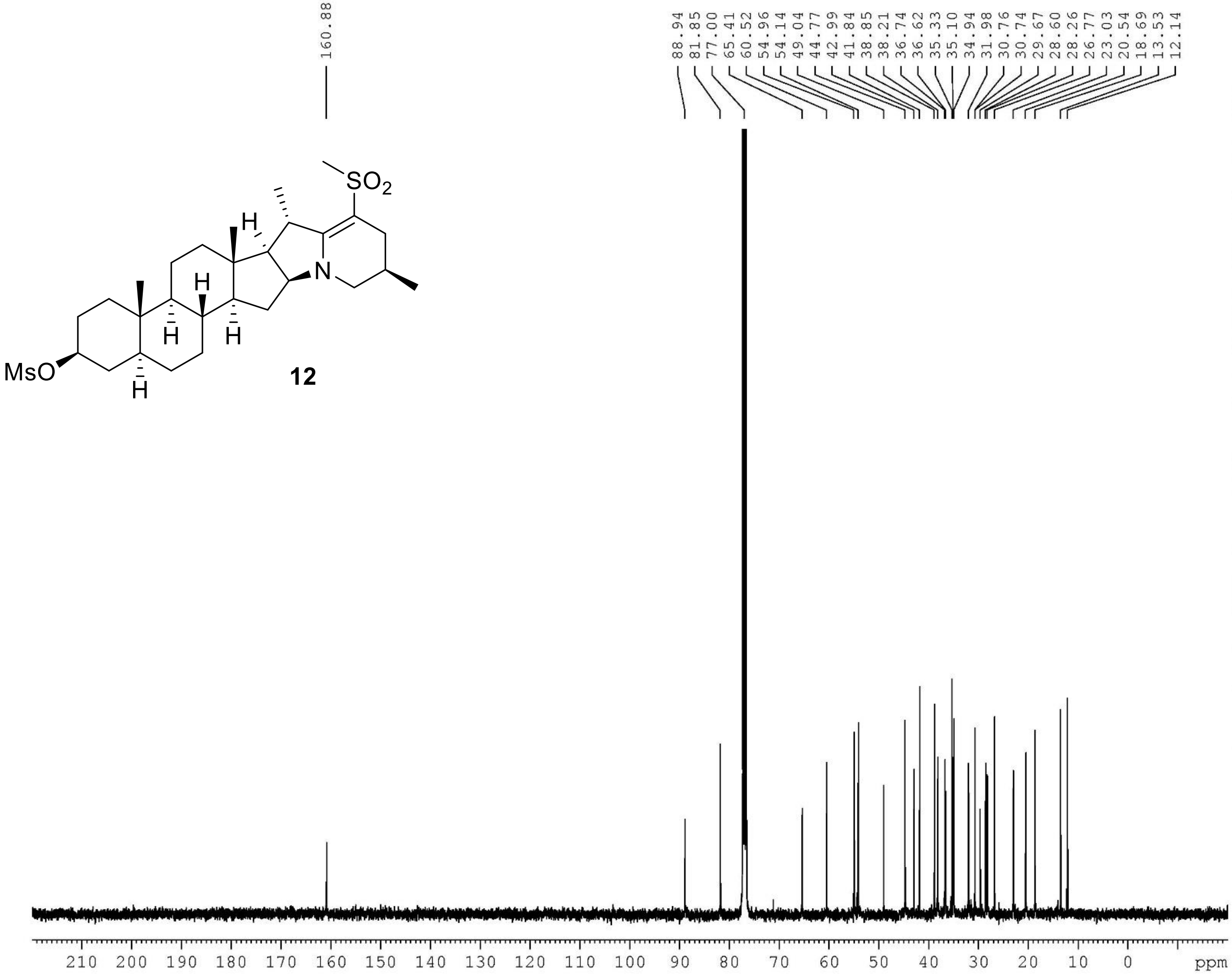


```
Current Data Parameters
NAME      AW G354b II (1)
EXPNO                  2
PROCNO                 1

F2 - Acquisition Parameter
Date_           20210402
Time                6.49
INSTRUM            spect
PROBHD   5 mm PABBO BB-
PULPROG          zgpg30
TD                 65536
SOLVENT            CDCl3
NS                 16384
DS                     4
SWH           32051.281 Hz
FIDRES         0.489064 Hz
AQ            1.0224116 se
RG                   101
DW               15.600 us
DE                 6.00 us
TE                299.1 K
D1          2.00000000 se
d11         0.03000000 se
DELTA       1.89999998 se
TD0                    1

======== CHANNEL f1 ======
NUC1                 13C
P1                 27.50 us
PL1                -1.00 dB
SFO1        100.6298721 MH

======== CHANNEL f2 ======
CPDPRG2           waltz16
NUC2                   1H
PCPD2             100.00 us
PL2                -3.00 dB
PL12               13.65 dB
PL13               18.00 dB
SFO2        400.1516006 MH

F2 - Processing parameters
SI                 32768
SF          100.6177993 MH
WDW                   EM
SSB                    0
LB                  1.00 Hz
GB                     0
PC                  0.20
```

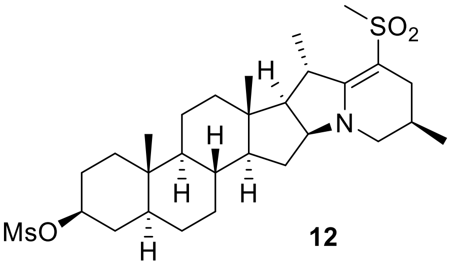
SO2
H
H
N
H
H
MsO
H

12

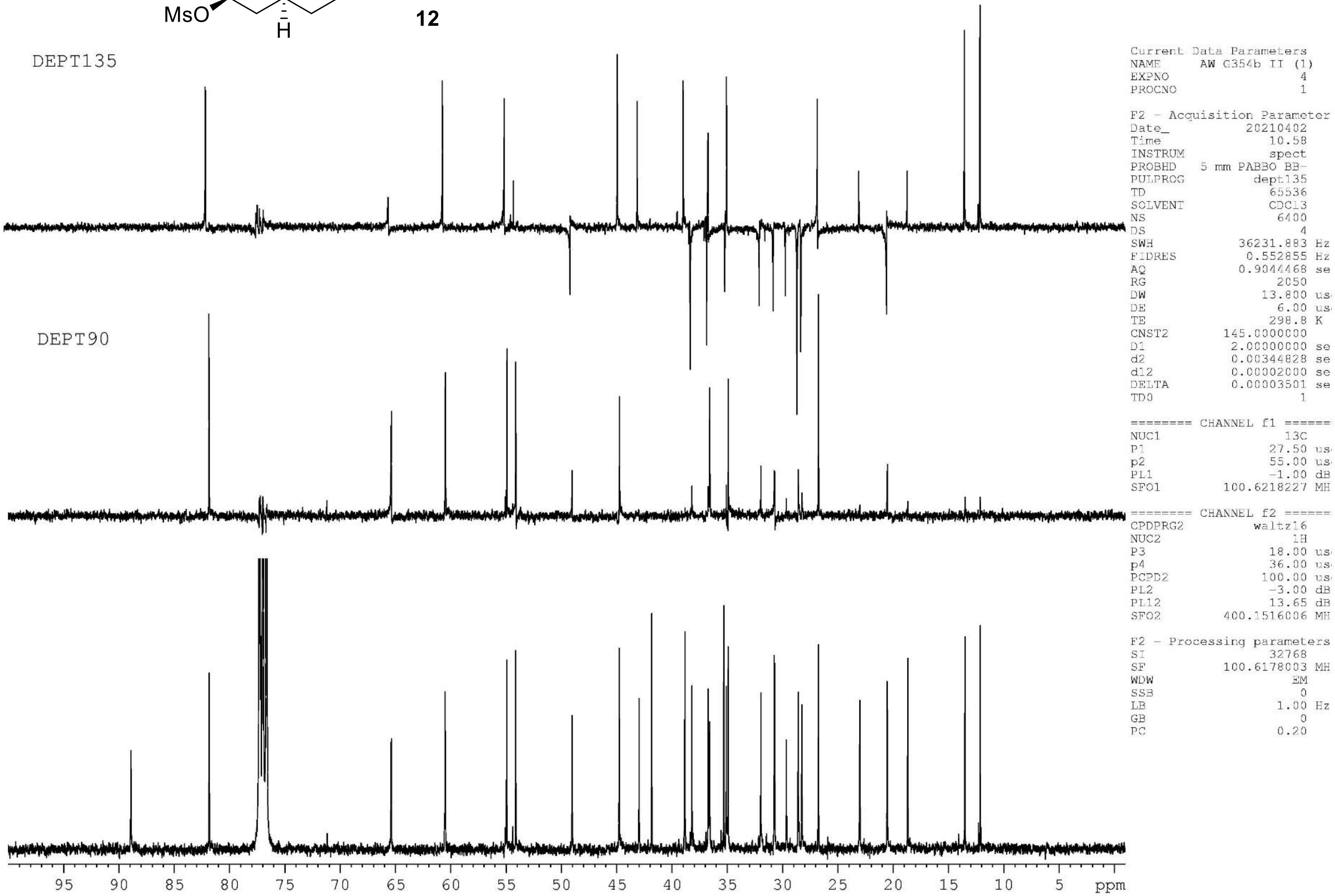
DEPT135
DEPT90
95 90 85 80 75 70 65 60 55 50 45 40 35 30 25 20 15 10 5 ppm
Current Data Parameters
NAME AW G354b II (1)
EXPNO 4
PROCNO 1
F2 - Acquisition Parameter
Date_ 20210402
Time 10.58
INSTRUM spect
PROBHD 5 mm PABBO BB-
PULPROG dept135
TD 65536
SOLVENT CDCl3
NS 6400
DS 4
SWH 36231.883 Hz
FIDRES 0.552855 Hz
AQ 0.9044468 se
RG 2050
DW 13.800 us
DE 6.00 us
TE 298.8 K
CNST2 145.0000000
D1 2.00000000 se
d2 0.00344828 se
d12 0.00002000 se
DELTA 0.00003501 se
TD0 1
======== CHANNEL f1 ======
NUC1 13C
P1 27.50 us
p2 55.00 us
PL1 -1.00 dB
SFO1 100.6218227 MH
======== CHANNEL f2 ======
CPDPRG2 waltz16
NUC2 1H
P3 18.00 us
p4 36.00 us
PCPD2 100.00 us
PL2 -3.00 dB
PL12 13.65 dB
SFO2 400.1516006 MH
F2 - Processing parameters
SI 32768
SF 100.6178003 MH
WDW EM
SSB 0
LB 1.00 Hz
GB 0
PC 0.20
